# Cyclic γ-Peptides With Transmembrane Water Channel Properties

**DOI:** 10.3389/fchem.2020.00368

**Published:** 2020-04-30

**Authors:** Jie Chen, Qiang Li, Pengchao Wu, Juan Liu, Dan Wang, Xiaohong Yuan, Renlin Zheng, Rongqin Sun, Liangchun Li

**Affiliations:** ^1^School of Life Science and Engineering, Southwest University of Science and Technology, Mianyang, China; ^2^School of Materials Science and Engineering, Southwest University of Science and Technology, Mianyang, China

**Keywords:** cyclic peptide, self-assembling, nanotube, hydrophobic inner cavity, water channels

## Abstract

Self-assembling peptides can be used to design new materials for medical and biological applications. Here we synthesized and characterized two novel cyclic γ-peptides (γ-CPs) with hydrophobic inner surfaces. The NMR and FT-IR studies confirmed that the CPs could self-assemble into parallel stacking structures via intermolecular H-bonds and π-π interactions. The morphologies of the self-assembly CPs showed bundles of nanotubes via transmission electron microscopy (TEM); these nanotubes form water channels to transport water across the lipid membrane. The properties of blocking the transport of protons like natural water channels showed that the hydrophobic inner surfaces are important in artificial transmembrane water channel designs. These studies also showed that water transport was a function of pore size and length of the assemblies.

## Introduction

Artificial compounds can form channels for ions and water that mimic natural channels (Sakai and Matile, 2013; Si et al., 2015; Barboiu, 2016; Huo and Zeng, 2016). These synthetic channels often contain structural motifs from biological assemblies including peptides (Hille, 2001; Hsieh and Liaw, 2019), DNA (Langecker et al., 2012), and steroids (Chen et al., 2005). Recent studies have shown that the integration of these assemblies with non-covalent forces, such as hydrogen bonding, electrostatic forces, hydrophobic and π-π interactions, and metal-organic architectures, can produce various synthetic channels and pores. Nevertheless, tools to tightly control the selectivity of the water or ions across the lipid membrane remain scarce (Si et al., 2015; Barboiu, 2016; Huo and Zeng, 2016; Gong, 2018).

Versus biological channels, most synthetic pores are hydrophilic with poor ion selectivity; only a few have demonstrated water selectivity. Pores with a diameter of ~3 Å are a critical requirement for ion-exclusion properties [e.g., I-quarter (Le Duc et al., 2011) and aquafoldamer (Fu et al., 2013; Zhao et al., 2014)] such pores can mimic the functions of natural Aquaporins (AQPs). Percec et al. recently showed that aquapores can transport water and exclude ions except protons through bilayer membranes (Percec et al., 2004). They used dendritic dipeptides that self-assemble via enhanced peripheral π-stacking of aromatic dendrons to form stable helical pores (14.5 Å average diameter). The ion-exclusion phenomena in these aquapores are based on hydrophobic effects, which seem to be more important than steric constraints. However, little work has been done since then to harness these hydrophobic effects in artificial transmembrane water channels.

Self-assembling peptides are critical to the design of new materials for medical and biological applications (Abbas et al., 2017; Li et al., 2017; Zou et al., 2017; Otter and Besenius, 2019). Self-assembling cyclic peptide nanotubes (SCPNs) are tubular supramolecular aggregates obtained by stacking flat cyclic peptides (Bong et al., 2001; Rodriguez-Vazquez et al., 2017; Silk et al., 2019; Song et al., 2019). One of their key features is precise control of their internal diameter; the properties of their outer surface can also be tuned via the constituent amino acids (Chapman et al., 2012; Lamas et al., 2018; Mendez-Ardoy et al., 2018; Shaikh et al., 2018). Most SCPNs have a hydrophilic inner cavity to transport hydrophilic molecules (Brea et al., 2010). However, the ion transport properties of a partially hydrophobic nanotube channel like α, γ-CP have also been described by Granja (Montenegro et al., 2013).

**Figure 1:**
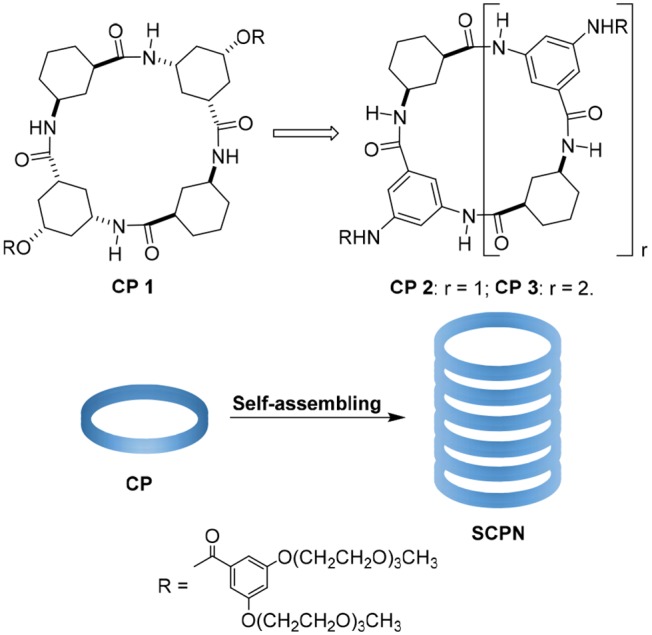
Peptide nanotube models formed by stacking of cyclic peptides (SCPN) and structures of cyclic peptides **1**, **2**, and **3**.

The tubular assemblies of **1** can self-assemble into nanotubes through hydrogen-bond-mediated parallel stacking, and this work further studies and optimizes their structure/function relationship (Li et al., 2012). Unfortunately, the structure of **1** is not amenable to extensive changes in the inner pore diameter because the γ-Ach is rigid and the corresponding molecules is not flat enough and favors the tetramer; this makes it difficult to obtain larger pores. In addition, the ester side chains are not stable enough for applications in physiological environment. Gong's designs are another tube system and can form hexamers when selecting the 1,3-disubstitued benzene residues as basic building blocks (Wu et al., 2015). Inspired by these studies, we designed two cyclic peptides by replacing the 3-amino-5-hydroxycyclohexane-1-carboxylic acid with 3,5-diaminobenzoic acid (Figure 1). After preparing the materials, we studied their transport properties. **2** is a tetrameric γ-peptide with an approximate Van der Waals internal diameter of 4 Å. **3** is a hexameric CP with an internal diameter of 8 Å that was used with **2** to test the effect of pore diameter on transport properties.

## Results and Discussion

**Scheme 1:**
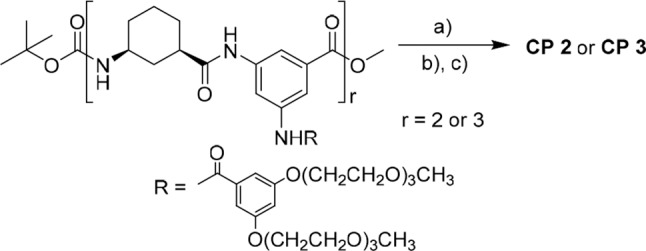
Synthesis of **2** and **3**: a) LiOH, MeOH/THF/H_2_O (1:1:1, v/v); b) HCl gas, DCM; and c) HATU, HOBT, DIPEA, DCM.

The prior solubility studies from our lab (Li et al., 2012; Lin et al., 2013) showed that the 3,5-diaminobenzoic acid was linked with 3,5-bis(triethylene glycol monomethyl ether)benzoate and contained two tri-EG (TEG) monomethyl ethers that improve peptide solubility. The integration of dendritic EG chains and hydrophobic cyclic peptides in the amphiphilic nanotubes can control the width and the length of the nanofibers that aggregate via β-sheet-like hydrogen-bonding between the cyclic hexapeptides of the hybrids. Thus, after removal of the N-terminal and C-terminal groups of **4a** and **4b**, the synthetic linear tetramer and hexamer were finally cyclized to form **2** and **3** that were then purified by column chromatography for 67 and 41% yields, respectively (Scheme 1).

**Figure 2:**
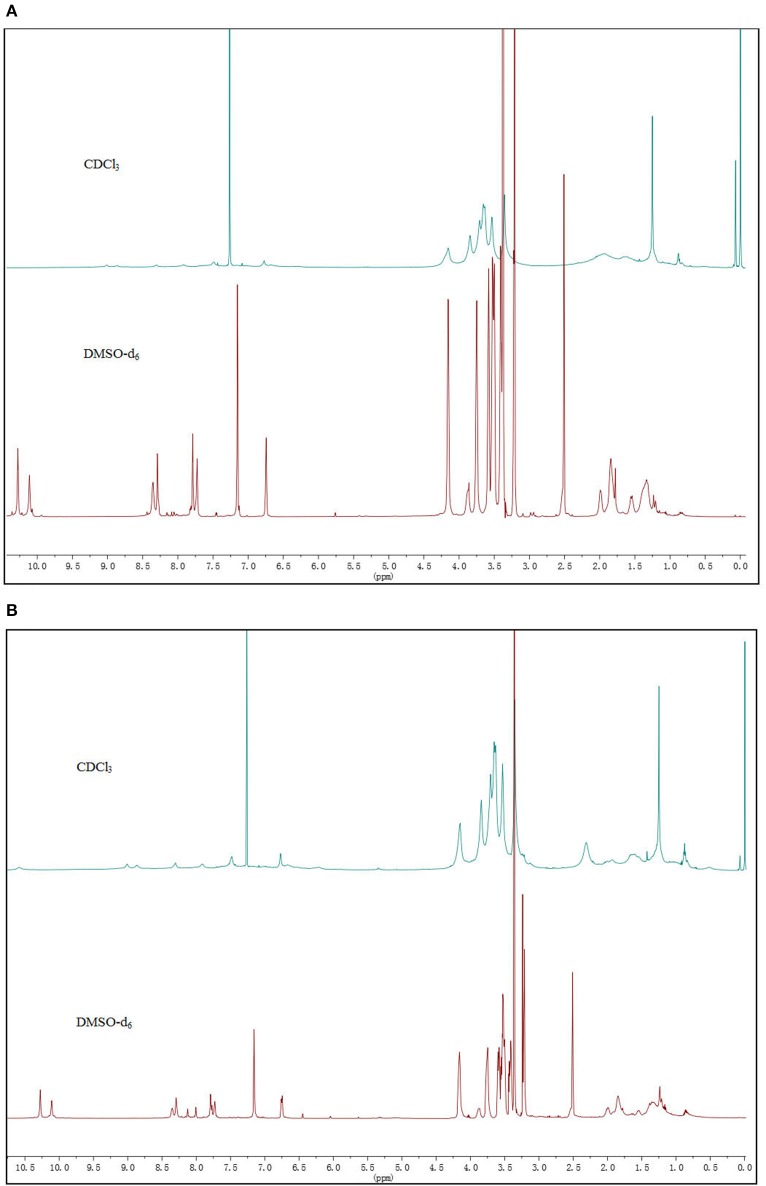
The ^1^H NMR spectra of CPs in DMSO and CDCl_3_: **(A) 2** and **(B) 3**.

**Figure 3:**
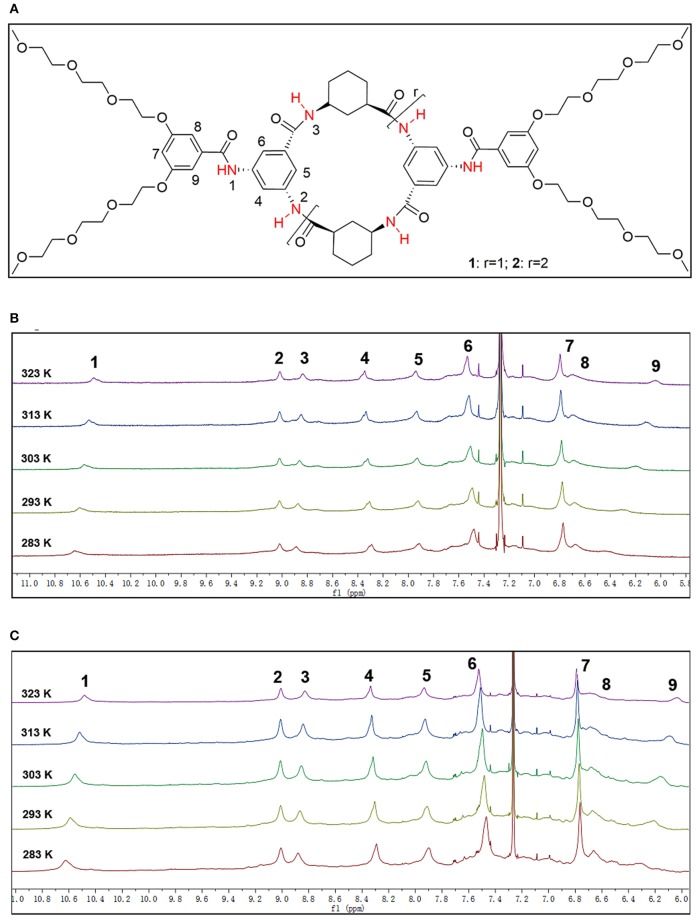
**(A)** The structure of the CPs, and the select 6.0–11.0 ppm region of variable temperature ^1^H NMR spectrum of **(B) 2** and **(C) 3** in CDCl_3_ (2 mM) showing the downfield shift of Ph-H and NH signals [NH signals (about 8.80–10.80 ppm) and Ar-H signals (about 6.00–8.4 ppm)].

The ^1^H NMR spectra of **2** and **3** displayed sharp and well-resolved signals in DMSO-d_6_ (Figure 2). In CDCl_3_, the broad and poorly-resolved peaks suggest self-assembly of cyclic peptides in the non-polar solution. The ^1^H NMR spectra in CDCl_3_ have three amide signals (δ = 8.8, 9.0 and 10.6 ppm) indicating that the 4 or 6 residue peptide adopts a symmetrical conformation. The downfield chemical shifts suggest that the proton is hydrogen bonded with the carbonyl group. Variable concentration (12 to 1 mM) experiments were performed on **2** and **3** but only a small upfield shift was observed for the NH proton (see Figures S4-2, S4-4 in Supporting Information); the variable temperature experiments in CDCl_3_ (2 mM) showed that the signals of the NH proton were shifted upfield by almost 0.1 ppm from 10 to 50°C for both **2** and **3** (see label 1 and 3 of N-H in Figure 3), which implies that the amide formed a stable intermolecular hydrogen-bonding (Brea et al., 2007). Furthermore, Ar-H resonance in the ring (label 9 in Figure 3) was upfield shifted from δ 6.43 to 6.04 as the temperature increase, while Ar-H resonance in the side-chain (label 4 in Figure 3) was downfield shifted from δ 8.29 to 8.35. Thus, the variable temperature experiments also showed that some Ar-H signals were shifted downfield or upfield as the temperature varied, implying that the aryl groups formed intermolecular π-π stacking aggregates.

The UV/Vis absorption spectra of CPs were measured in DCM at room temperature with different concentrations. Species **2** and **3** exhibited strong UV/Vis absorption at about 250 nm, which is due to the π-π^*^ transition of the aryl moieties (Perkampus, 1992). The intensity increased as the concentration varied from 0.02 to 1 mM. However, it is difficult to identify the absorption bands and research the π-π stacking aggregates via signal shifting.

**Figure 4:**
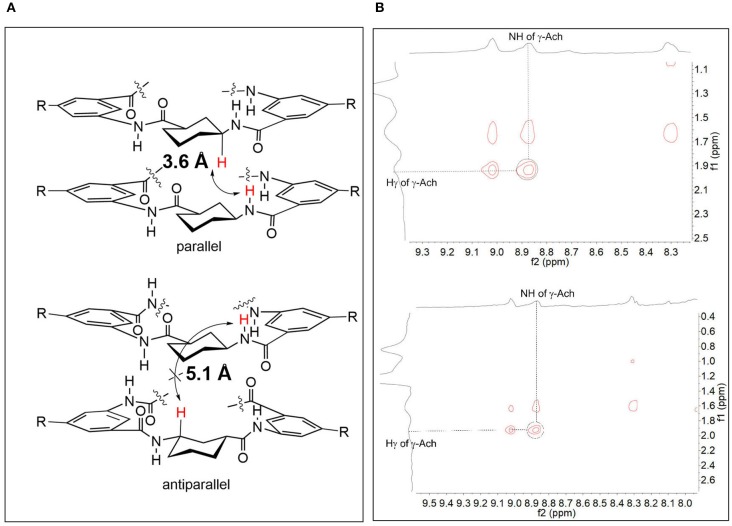
**(A)** The arrow shows the NH protons of γ-Ach and the Hγ of γ-Ach in the parallel stacking dimer and in the antiparallel stacking dimer (both calculated dimers of **2** and **3** are almost the same and the repeat structures are cut for clarify); **(B)** Selected region of the NOESY spectra of **2** (top) and **3** (bottom) in CDCl_3_.

We next performed further DFT calculations on **2** and **3** to study the conformations of the CPs (Frisch et al., 2009). The results confirmed that the parallel stackings of **2** and **3** are much more energetically favorable than the antiparallel ones. The antiparallel stacking of **2** is not stable in solution (similar to 1, Table S13). The calculations showed that the distances between the benzene rings in the parallel stacking conformation were <5 Å (about 4.7–4.8 Å, see Figure S13-2 in Supporting Information), which support the presence of π-π stacking interactions. We also determined that the distances of the NH protons of γ-Ach and the Hγ of γ-Ach in the parallel stacking dimers (about 3.6 Å) are closer than those in the antiparallel stacking dimers (about 5.1 Å) (Figure 4A). We then performed NOESY experiments to verify whether NOE cross-peaks exist. Figure 4B shows NOE cross peaks between NH protons of γ-Ach (about 8.85 ppm) and the Hγ of the γ-Ach (about 1.92 ppm) in both **2** and **3**. Such peaks are consistent with the proposed parallel stacking structure.

A solution of **2** and **3** in CHCl_3_ (2 mM) was equilibrated against n-hexane via vapor-phase diffusion. A colorless gel was formed after incubating at room temperature for 3–4 days. Fourier transform infrared (FT-IR) studies of the dried gel deposited on KBr pellets showed substantial evidence for the self-assembly of the peptides; the spectroscopy of **2** and **3** (Figure S6 in Supporting Information) are nearly identical. The spectra showed sharp peaks at 1,647 cm^−1^ in the amide I region and 1,596 cm^−1^ in the amide II region. These are characteristic of extensive hydrogen-bonding β-sheet-like networks. Moreover, the broad signal at 3,307 cm^−1^ also corresponds to the tightly hydrogen bonded ring-stacked networks (Bong et al., 2001; Rodriguez-Vazquez et al., 2017). These results suggest that the gels are constructed from tightly packed nanotubes formed during self-assembly of CPs; the basis for these nanotubes is β-sheet-like hydrogen-bonding. In the meantime, the absence of signal near 1,690 cm^−1^ corresponding to antiparallel β-sheet in **2** or **3** further suggests parallel β-sheet structures in these compounds (Clark et al., 1998; Li et al., 2012).

**Figure 5:**
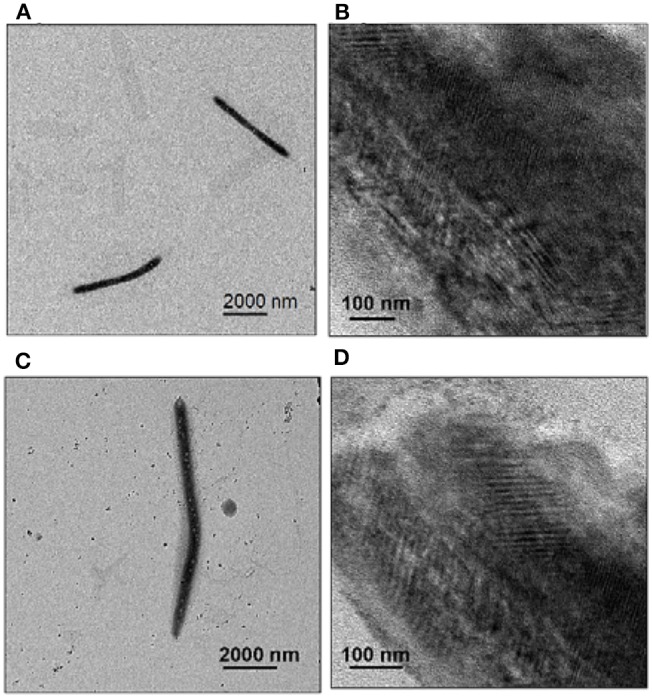
TEM images of gels formed by **2 (A,B)** and **3 (C,D)**. Each compound (2 mM in CHCl_3_) was equilibrated against n-hexane via vapor-phase diffusion for several days to obtain the gels for sampling. TEM images were obtained after the gels were dispersed in n–hexane.

The organogel was then dried and dispersed in n-hexane by sonication for transmission electron microscopy (TEM). TEM images of **2** confirmed that the nanofibers were 250 nm wide and 4–5 μm long (Figure 5A). This implies the formation of nanotube bundles because a single CP nanotube is about 5 nm wide. Some of the thin nanofibers were about 6 nm wide and 100–200 nm long (Figure 5B), which implied side-by-side packing of single nanotubes. Similarly, TEM images of assembly organogel samples formed by **3** showed that nanofibers had a width of 300–350 nm and length of 9–10 μm (Figure 5C); the thin nanofibers were about 10–14 nm width and 400–500 nm long (Figure 5D). The fibers formed by **3** were longer than those formed by **2**.

**Figure 6:**
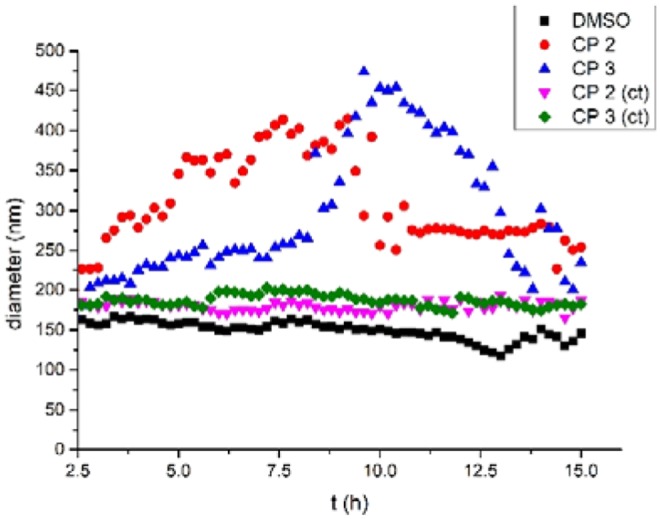
Mean diameter of the vesicles with time after addition of a DMSO solution of CP **2**, **3** (0.3 mol% relative to lipid) or pure DMSO.

After confirming tube formation, we next evaluated their functions. The water transport properties of the tubular structures across lipid bilayers were investigated using dynamic light scattering (DLS) (Hallett et al., 1993). Large unilamellar vesicles in buffer containing NaCl (100 mM) were first prepared from egg yolk L-α-phosphatidylcholine (EYPC) and then suspended in pure water to produce different osmotic pressures inside and outside the vesicles. Aliquots of DMSO solutions of **2** and **3** (0.3% molar ratio relative to lipid) were injected into the vesicle suspensions. The light scattering and vesicle diameters were then monitored for 15 h. Figure 6 shows that adding the solution of **2** and **3** in DMSO caused the mean vesicle diameter to increase from ca. 180 nm to ca. 450 nm within 9 h. In contrast, adding pure DMSO did not lead to a detectable influence.

When the vesicles were suspended in buffer containing NaCl (100 mM), the osmotic pressure was the same inside and outside the vesicles. Adding **2** and **3** to the suspension did not change the size of the vesicles within 15 h (CP **2** (ct) and CP **3** (ct) in Figure 6). This implies that **2** and **3** did not promote vesicle fusion. Thus, based on prior work about water channels (Hu et al., 2012), we suspect that the vesicle increase is due to water transport from outside the vesicles to the inside via osmotic pressure differences. The transport of water was detected after almost 2.5 h, which is quite late vs. other artificial water channels. This suggests that the formation of cyclic peptide tubes is slow in membranes. However, the size of the vesicles increased 2-fold within 7 h for **2** and 9 h for **3**, which implied that the tubes formed by **2** were more efficient than **3** in membranes. After 9–11 h, the vesicle diameters decreased gradually because of the precipitation of large vesicles.

**Figure 7:**
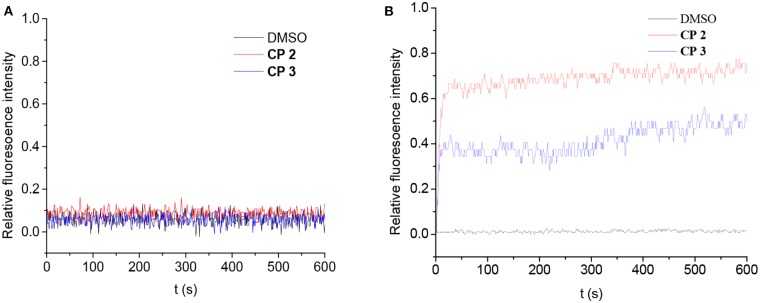
Change in fluorescence intensity of vesicles (addition of DMSO, CP **2** and **3**). The vesicles were loaded with NaCl and HPTS buffered at pH 7.0 with HEPES and suspended in KCl: **(A)** buffered to pH 5.5; **(B)** buffered to pH 7.6.

The transport-originating properties of **2** and **3** were then evaluated for the H^+^/OH^−^ transport (Figure 7) using a pH gradient across the vesicle membranes (Jeon et al., 2004). Large unilamellar vesicles were prepared from egg yolk L-R-phosphatidylcholine (EYPC) with and without **2** or **3** (1 mol %) as described in the literature. The H^+^/OH^−^ flux through the membranes was assessed by changes in fluorescence intensity of the pH-sensitive dye 8-hydroxypyrene-1,3,6-trisulfonate (HPTS) entrapped inside the vesicles. A suspension of vesicles entrapping the HPTS and HEPES buffer (pH 7.0 inside the vesicles) was prepared and **2** or **3** in DMF (5.0 mM, 20.0 μL) were added with gentle stirring. We then added these vesicles to HEPES buffers with a pH of 5.5 or 7.6 to produce higher H^+^ or OH^−^ concentrations outside the vesicles.

Compounds **2** and **3** had no change in fluorescence intensity of HPTS at pH 5.5, suggesting that they did not transport protons across the membrane. However, there was OH^−^ flux through the membrane. Adding **2** or **3** to the vesicle suspension in higher OH^−^ buffer changed the fluorescence intensity. One of the key features of natural water channels is blocking of the proton flux; these results suggest that the cyclic peptides act as natural water channels (Jensen et al., 2005). Furthermore, **2** exhibited higher transport ability, which implies that the bigger diameter of the cyclic peptide does not improve the OH^−^ transport rate but does improve the water diffusion ability (Possibly due to the shorter length of nanaotubes formed by **2**). It is interesting that the results of blocking proton flux were different from Granja's CP, which suggested the formation of the proton-transport channel (Brea et al., 2010; Rodriguez-Vazquez et al., 2016).

## Conclusion

In summary, we designed and synthesized two cyclic γ-peptides with hydrophobic inner surfaces. The NMR, IR, and TEM showed that CPs **2** and **3** could aggregate to form nanotubes via intermolecular stacking. The CPs form selective water channels that block H^+^ flux to transport water across the lipid membrane. Prior work has shown that proton transport was not blocked by the cyclic peptide nanotubes (including hydrophilic inner surfaces and Granja's partial hydrophobic inner surfaces of CPs) (Brea et al., 2010; Chapman et al., 2012). Thus, these results show that the fully hydrophobic inner surfaces affect the behavior of cyclic peptide nanotubes in transport properties. These cyclic peptide systems may have utility for biomimicking water channels.

## Data Availability Statement

All datasets generated for this study are included in the article/Supplementary Material.

## Author Contributions

LL designed the study and wrote the protocol. JC undertook the main experimental works, preformed the SEM/TEM analysis, managed the literature search and wrote the first draft of the manuscript with assistance from RS. QL preformed the ion channel experiments and synthetic works with the assistance from XY. PW did part of synthetic works about the cyclic peptides with assistance from DW. JL did part of synthetic works with assistance from RZ.

## Conflict of Interest

The authors declare that the research was conducted in the absence of any commercial or financial relationships that could be construed as a potential conflict of interest.
