# Supplementary material for: Cyclic γ-Peptides With Transmembrane Water Channel Properties
Source: Front Chem. 2020 Apr 30;8:368. doi: 10.3389/fchem.2020.00368 (PMC7205449; doi:10.3389/fchem.2020.00368)
Supplement: Supplementary file 1 [file Data_Sheet_1.pdf]

Supporting Information

Cyclic γ-peptides with transmembrane water channel properties

*Jie Chen, Qiang Li, Pengchao Wu, Juan Liu, Dan Wang, Xiaohong Yuan, Renlin Zheng, Rongqin Sun, Liangchun Li**

Contents:

1. General Methods, Instrument Details and Materials

2. Synthesis

3. ^1H NMR and ^{13}C NMR spectra

4. The variable-concentration and variable-temperature ^1H NMR spectra of cyclotetrapeptide **2** and cyclohexapeptide **3**

5. HSQC and NOESY spectra of **2** and **3** in $CDCl_3$

6. The FT-IR spectra of **2** and **3**

7. MALDI-TOF Results

8. The UV-vis spectra of **2** and **3** dissolved in dichloromethane with different concentration

9. Procedures for OH^- transport experiments

10. Procedures for H^+ transport experiments

11. Gels images of **2** and **3** assemblies

12. SEM and TEM images of **2** and **3** assemblies.

13. DFT calculations

1. General Methods, Instrument Details and Materials

General

γ-Ach and γ-Ach protected with TEG were prepared with previous reports of our lab[1]. O-(7-azabenzotriazol-1-yl)-1,1,3,3-tetramethyluronium hexafluorophosphate (HATU) , 1-Hydroxybenzotriazole (HOBt) , N,N-Diisopropylethylamine (DIPEA) and 4-Dimethylaminopyridine (DMAP), 8-hydroxypyrene-1,3,6-trisulonate (HPTS) were all used as obtained from Energy Chemical (Shanghai) Ltd. Bis-N-methylacridinium nitrate (Lucigenin) and Acetylcholine chloride were obtained from Tokyo Chemical Industry (Shanghai) Co. Ltd. N-(3-dimethylaminopropyl)-N-ethylcarbodiimide hydrochloride (EDCI) , Egg yolk L-α-phosphatidyl-choline (EYPC, 5g), 2-(N-Morpholino)ethanesulfonic acid (MES), 2-[4-(2-Hydroxyethyl)-1-pipera zinyl]ethanesulfonic acid (HEPES), Triton X-100 were all obtained from Aladdin Industrial Corporation (Shanghai) Ltd. All other reagents obtained from commercial suppliers were used without further purification unless otherwise noted. Dichloromethane (CH_2Cl_2) was dried and distilled over calcium hydride. Silica gel flash chromatography was performed using silica gel (400-500 mesh) from Qingdao Haiyang Chemical Co. Ltd.

NMR experiments. Proton nuclear magnetic resonance (^{1}H NMR) spectra were recorded on Bruker WM-600 MHz spectrometers. Chemical shifts were reported in parts per million (ppm, δ) from TMS or solvent resonance as the internal standard. Carbon nuclear magnetic resonance (^{13}C NMR) spectra were recorded on Bruker WM-600 MHz spectrometers.

Matrix Assisted Laser Desorption Ionization-Time of Flight (MALDI-TOF) was obtained on a Bruker Autoflex mass spectrometer.

Electrospray (ESI) mass spectra were recorded on a Bruker BioTOF Q mass spectrum.

FT-IR measurements were made on a Spectrum one (Version BM) spectrophotometer by the KBr method.

UV/Vis spectra were recorded on UV5500PC spectrophotometer and the sample were dissolved by dichloromethane.

2. Synthesis

2.1 Synthesis methods of γ-ACHC

The synthesis methods of γ-ACHC were showed as Scheme S1, which were prepared with previous reports [1].

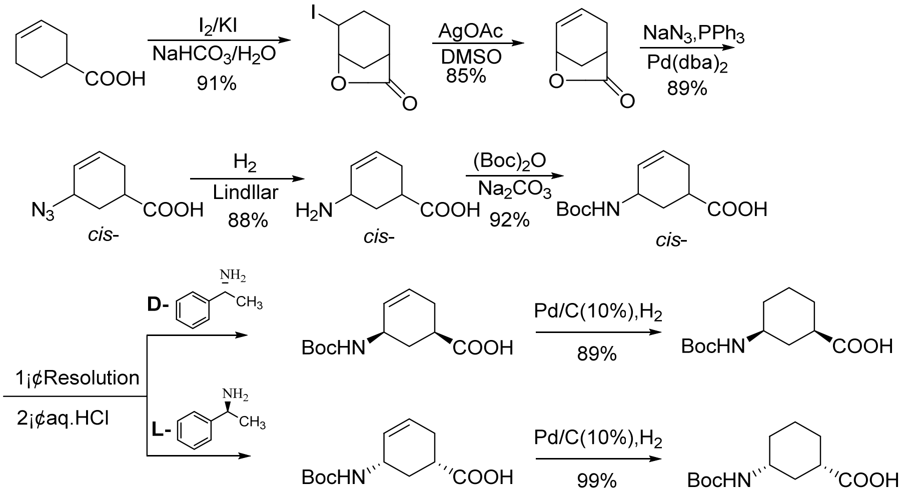

Scheme S2-1 The synthesis of γ-ACHC

(1R,3S)-γ-Ach (100%ee): ^{1}H NMR (300 MHz, $CDCl_3$) δ (ppm): 4.49 (d, *J* = 7.0 Hz, NH), 3.51-3.33 (m, 1H), 2.44-2.27 (m, 1H), 2.00-1.85 (m, 3H), 1.46 (s, 9H), 1.38-1.09 (m, 4H).

2.2 Synthesis methods of Cyclic peptide side chain

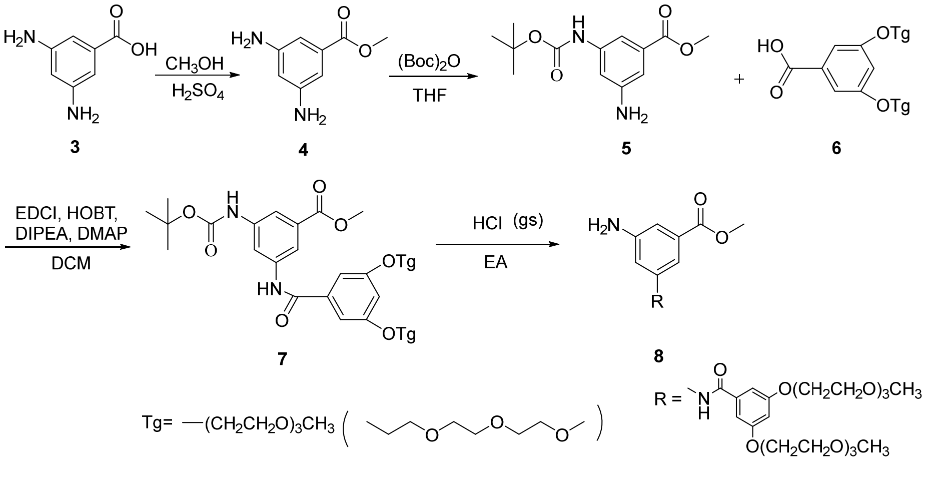

Scheme S2-2 The synthesis of **8**

The synthesis procedures:

Compound 4：3,5-Diaminobenzoic Acid **3** (15.2 g, 100 mmol) was suspended in MeOH (180 mL). Sulfuric acid (3 mL, 368 mmol) was slowly added and the solution

was in an ice bath. Then stirred and refluxed at 90 ºC for 6 hours. After cooling, the solution was adjusted the pH to 8, extracted with ethyl acetate, the extract was dried over anhydrous Na_2SO_4. Then, after filtration of the solution, the solvent was removed and get target product **4** (15.744 g, 94.8%) as a brown solid.

^{1}H NMR (600 MHz, DMSO-d_6) δ 6.43 (d, *J* = 2.0 Hz, 2H), 6.03 (t, *J* = 2.0 Hz, 1H), 5.00 (s, 4H), 3.74 (s, 3H).[2]

Compound 5：To a solution of **4** (1.66 g, 10 mmol) in 25 mL of THF at 0 ºC, was slowly added triethylamine (2.1 mL, 15 mmol). Then di-tert-butyl dicarbonate (2.4 mL, 10.5 mmol) was dissolved in THF (25 mL) and added to the solution that was stirred for 22 h at room temperature. After stirring the resulting solution until completion of the reaction, the solution was concentrated in vacuum. The residue was resolved in ethyl acetate and washed with 1M HCl(aq). Then the aqueous layer was adjusted the pH to 8-9 with solution of sodium carbonate and extracted with ethyl acetate (150 ml). The organic phase was dried over anhydrous Na_2SO_4 and concentrated in vacuum and the mixture was purified by column chromatography (PE:EA=2:1) and get **5** (1.6 g, 60.2%) as a brown solid.

^{1}H NMR (600 MHz, $CDCl_3$) δ 7.24 (s, 1H), 7.19 (s, 1H), 7.02 (s, 1H), 6.59 (s, 1H), 3.87 (s, 3H), 3.86 – 3.59 (m, 2H), 1.51 (s, 9H).

Compound 7：To a solution of **6** (4.46g, 10 mmol), EDCI (3.83 g, 20 mmol), HOBt (2.70 g, 20 mmol) and DMAP (1.22 g, 10 mmol) in CH_2Cl_2 (100 mL) at -10-0 ºC for 1 h, were added **5** (2.66 g，10 mmol) dissolved in CH_2Cl_2 (25 mL) and DIPEA (6.6 mL, 40 mmol). Then the mixture was warmed up to room temperature slowly and stirred for 48 h at room temperature. After stirring the resulting solution until completion of the reaction, 1M HCl (aq) was poured into the mixture and the product was extracted with CH_2Cl_2 . The combined organic layers were washed with 3N $NaHCO_3$ (aq), water and brine. Then the organic phase was dried over anhydrous Na_2SO_4 and concentrated in vacuum, and the mixture was purified by column chromatography (PE:EA=1:8) and get **7** (5.440 g, 78.4%) as a light yellow oil.

^{1}H NMR (600 MHz, $CDCl_3$) δ 8.89 (s, 1H), 8.21 (s, 1H), 8.09 (s, 1H), 7.88 (s, 1H), 7.28 (d, *J* = 2.8 Hz, 1H), 6.92 (d, *J* = 2.1 Hz, 2H), 6.53 (t, *J* = 2.1 Hz, 1H), 4.10 (dd, *J* = 11.2, 6.9 Hz, 4H), 3.89 (s, 3H), 3.86 – 3.82 (m, 4H), 3.76 – 3.72 (m, 4H), 3.69 (dd, *J* = 5.8, 3.5 Hz, 4H), 3.64 (dd, *J* = 5.5, 3.8 Hz, 4H), 3.53 (dd, *J* = 5.6, 3.7 Hz, 4H), 3.33 (s, 6H), 1.48 (s, 9H).

Compound 8：Compound **7** (440 mg, 0.6 mmol) was dissolved in ethyl acetate (5 mL). HCl (g) is introduced into the solution and stirred for 15-20 min. Testing of raw materials reacted complete by TLC, and the solvent was removed under reduced pressure. Then the aqueous layer was adjusted the pH to 8-9 with solution of sodium carbonate and extracted with ethyl acetate (50 ml). The organic phase was dried over anhydrous Na_2SO_4 and concentrated in vacuum to get **8** (0.318 g, 84.4%) as a light yellow oil.

^{1}H NMR (600 MHz, $CDCl_3$) δ 8.59 (s, 1H), 7.59 (s, 1H), 7.50 (s, 1H), 7.08 (d, *J* = 11.3 Hz, 1H), 6.98 (d, *J* = 2.1 Hz, 2H), 6.55 (t, *J* = 2.1 Hz, 1H), 4.12 – 4.08 (m, 4H), 3.97 (d, *J* = 11.6 Hz, 2H), 3.86 (s, 3H), 3.84 – 3.81 (m, 4H), 3.75 – 3.71 (m, 4H), 3.68 (dd, *J* = 5.7, 3.5 Hz, 4H), 3.64 (dd, *J* = 5.6, 3.7 Hz, 4H), 3.53 (dd, *J* = 5.6, 3.8 Hz, 4H), 3.34 (s, 6H).

2.3 Synthesis methods of dipeptide (9) and tetrapeptide (10)

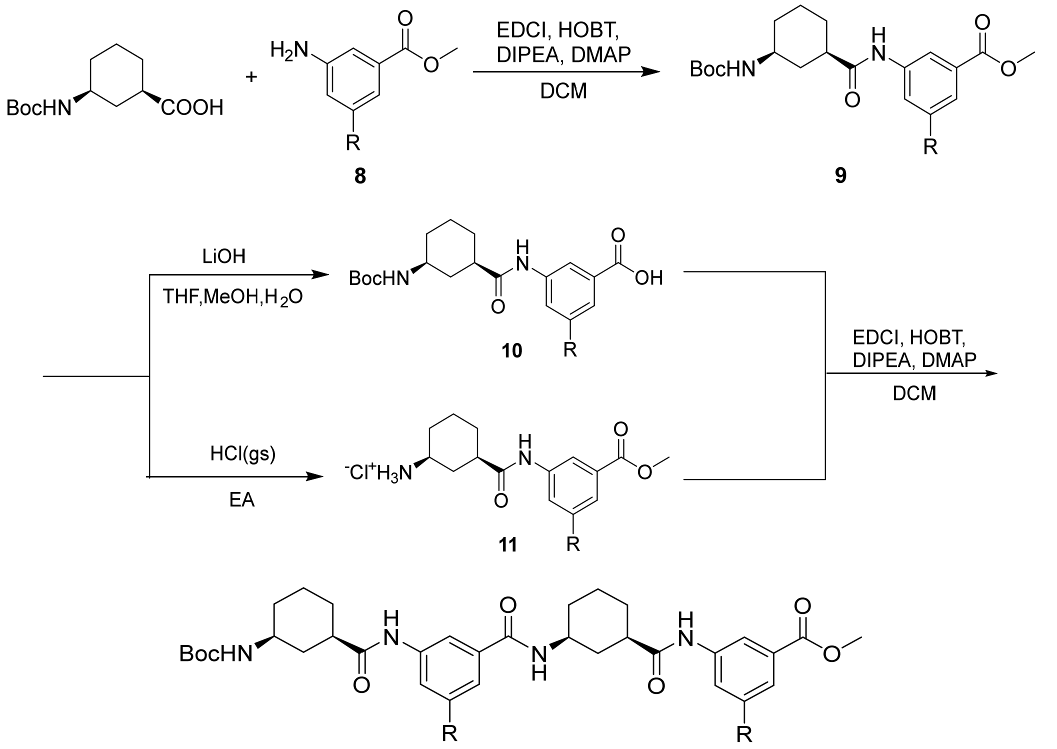

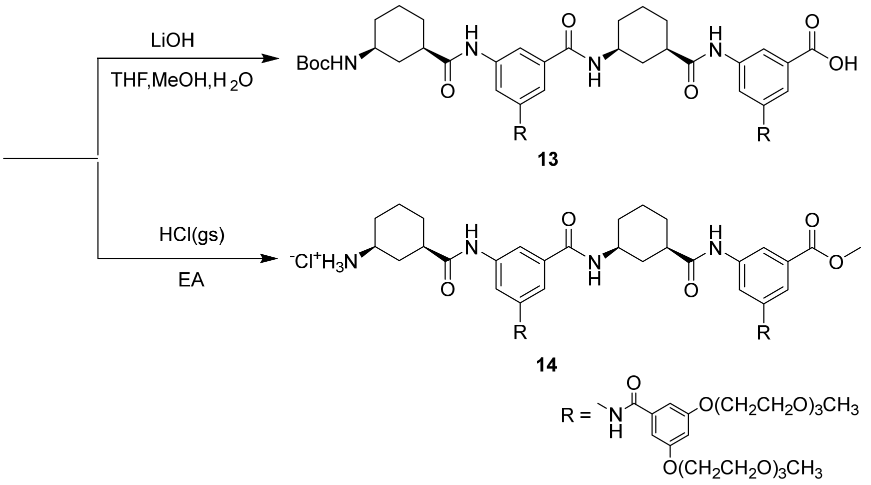

Scheme S2-3 The synthesis of tetrapeptide.

The synthesis procedures:

Dipeptide 9: To a solution of γ-**Ach** (1.16 g, 5 mmol), EDCI (1.92 g, 10 mmol), HOBt (1.35 g, 10 mmol) and DMAP (0.61 g, 5 mmol) in CH_2Cl_2 (50 mL) at -10-0 ºC for 1 h, were added **8**(2.86 g, 5 mmol) dissolved in CH_2Cl_2 (50 mL) and DIPEA (3.3 mL, 20 mmol). Then the mixture was warmed up to room temperature slowly and stirred for 48 h at room temperature. After stirring the resulting solution until completion of the reaction, 1M HCl (aq) was poured into the mixture and the product was extracted with CH_2Cl_2. The combined organic layers were washed with 3N $NaHCO_3$ (aq), water and brine. Then the organic phase was dried over anhydrous Na_2SO_4 and concentrated in vacuum, and the mixture was purified by column chromatography (CH_2Cl_2:EA: Acetone=21:2:5) and get **9** (3.275 g, 82.7%) as a light yellow oil.

^{1}H NMR (600 MHz, $CDCl_3$) δ 9.27 (s, 1H), 8.60 (s, 1H), 8.39 (s, 1H), 8.29 (s, 1H), 7.95 (d, J = 11.4 Hz, 1H), 6.93 – 6.84 (m, 2H), 6.51 (d, J = 10.2 Hz, 1H), 4.63 (d, J = 7.7 Hz, 1H), 4.08 (t, J = 4.2 Hz, 4H), 3.88 (s, 3H), 3.84 (t, J = 6.7 Hz, 4H), 3.78 – 3.74 (m, 4H), 3.73 – 3.69 (m, 4H), 3.66 – 3.63 (m, 4H), 3.54 – 3.50 (m, 4H), 3.45 – 3.37 (m, 1H), 3.33 (s, 6H), 2.32 (t, J = 11.4 Hz, 1H), 2.14 (d, J = 4.7 Hz, 1H), 1.93 (d, J = 11.0 Hz, 1H), 1.84 – 1.75 (m, 2H), 1.45 (d, J = 6.0 Hz, 9H), 1.36 – 1.20 (m, 3H), 1.03 (dt, J = 9.0, 12.0 Hz, 1H).

^{13}C NMR (151 MHz, $CDCl_3$) δ 173.12, 165.75, 165.31, 158.75, 154.25, 138.31, 137.80, 135.81, 130.11, 116.03, 115.83, 115.17, 105.14, 103.70, 78.23, 70.85, 69.72, 69.63, 69.48, 68.61, 66.56, 57.92, 51.19, 48.11, 43.92, 35.00, 31.64, 28.68, 27.43, 27.16, 23.28.

Compound 10：Under a ice water condition, Compound **9** (0.336 g, 0.41 mmol) was dissolved in THF (5 mL) and MeOH (10 mL) mixed solvent and Lithium Hydroxide Monohydrate aqueous solution (0.086 g/5 mL) was added, keep this condition stirring for 0.5 hour and then moved to room temperature stirred overnight. Testing of raw materials reacted complete by TLC (CH_2Cl_2:EA: Acetone=21:2:5), and the solvent was removed under reduced pressure. The aqueous phase was then acidified with 1M HCl (aq) to pH 2, extracted with CH_2Cl_2, then the organic phase was dried over anhydrous Na_2SO_4 and concentrated in vacuumto get **10** (0.339 g, 102.7%) as a light yellow oil.

Compound 11：Compound **9** (169 mg, 0.2 mmol) was dissolved in ethyl acetate (10 mL). HCl (g) is introduced into the solution and stirred for 15-20 min. Testing of raw materials reacted complete by TLC (CH_2Cl_2:EA: Acetone=21:2:5), and the solvent was removed under reduced pressure. Then the aqueous layer was adjusted the pH to 8-9 with solution of sodium carbonate and extracted with ethyl acetate (50 ml). The organic phase was dried over anhydrous Na_2SO_4 and concentrated in vacuum to get **11** (0.113 g, 76.1%) as a light yellow oil.

Tetrapeptide 12: To a solution of **10** (0.124 g, 0.154 mmol), EDCI (0.059 g, 0.308 mmol), HOBt (0.042 g, 0.308 mmol) and DMAP (0.019 g, 0.154 mmol) in CH_2Cl_2 (5 mL) at -10-0 ºC for 1 h, were added **11** (0.111 g, 0.154 mmol) dissolved in CH_2Cl_2 (5 mL) and DIPEA (0.102 mL, 0.616 mmol). Then the mixture was warmed up to room temperature slowly and stirred for 48 h at room temperature. After stirring the resulting solution until completion of the reaction, 1M HCl (aq) was poured into the mixture and the product was extracted with CH_2Cl_2. The combined organic layers were washed with 3N $NaHCO_3$ (aq), water and brine. Then the organic phase was dried over anhydrous Na_2SO_4 and concentrated in vacuum, and the mixture was purified by column chromatography ($CHCl_3$:MeOH=50:1, TLC by CH_2Cl_2:MeOH:EA=13:1:3) and get **12** (0.200 g, 84.4%) as a colorless oil.

^{1}H NMR (600 MHz, DMSO-d_6) δ 10.35 (s, 1H), 10.27 (s, 1H), 10.21 (s, 1H), 10.04 (s, 1H), 8.44 (s, 1H), 8.35 (d, *J* = 8.0 Hz, 1H), 8.28 (s, 1H), 8.09 (s, 1H), 8.05 (s, 1H), 7.79 (s, 1H), 7.73 (s, 1H), 7.16 (s, 4H), 6.83 (d, *J* = 8.1 Hz, 1H), 6.78 – 6.74 (m, 2H), 4.20 – 4.15 (m, 8H), 3.87 (s, 3H), 3.77 (s, 8H), 3.60 (dd, *J* = 6.3, 2.9 Hz, 8H), 3.55 (dd, *J* = 7.0, 4.0 Hz, 8H), 3.53 – 3.51 (m, 8H), 3.44 –

3.42 (m, 8H), 3.34 – 3.27 (m, 1H), 3.23 (d, J = 1.0 Hz, 12H), 2.54 (d, J = 12.0 Hz, 1H), 2.43 (s, 1H), 2.01 (d, J = 12.7 Hz, 1H), 1.91 (d, J = 11.6 Hz, 1H), 1.85 (t, J = 10.8 Hz, 3H), 1.81 – 1.71 (m, 3H), 1.55 (q, J = 12.1 Hz, 1H), 1.47 – 1.21 (m, 16H), 1.16 – 1.07 (m, 1H).

^{13}C NMR (151 MHz, DMSO-d_6) δ 174.15, 173.97, 166.52, 166.34, 165.48, 165.29, 160.04, 155.30, 140.38, 140.12, 139.80, 139.48, 136.93, 136.85, 136.58, 130.57, 116.31, 115.92, 115.65, 115.24, 114.63, 114.49, 106.85, 106.76, 104.75, 77.93, 71.74, 70.42, 70.28, 70.08, 69.35, 67.98, 58.50, 52.70, 49.09, 48.23, 44.50, 44.46, 36.10, 35.49, 32.51, 32.12, 28.73, 28.56, 24.54, 24.49.

Compound 13：Under a ice water condition, Compound **12** (0.150 g, 0.1 mmol) was dissolved in THF (3 mL) and MeOH (6 mL) mixed solvent and Lithium Hydroxide Monohydrate aqueous solution (0.021 g/3 mL) was added, keep this condition stirring for 0.5 hour and then moved to room temperature stirred overnight. Testing of raw materials reacted complete by TLC (CH_2Cl_2:MeOH:EA=12:1:1), and the solvent was removed under reduced pressure. The aqueous phase was then acidified with 1M HCl (aq) to pH 2, extracted with EA, then the organic phase was dried over anhydrous Na_2SO_4 and concentrated in vacuumto get **13** (0.122 g, 82.1%) as a light yellow oil.

Compound 14：Compound **12** (68 mg, 0.05 mmol) was dissolved in ethyl acetate (10 mL). HCl (g) is introduced into the solution and stirred for 30-40 min. Testing of raw materials reacted complete by TLC (CH_2Cl_2:MeOH:EA=12:1:1), and the solvent was removed under reduced pressure to get **14** (0.060 g, 94.5%) as a light yellow solid for subsequent synthesis.

2.4 Synthesis methods of CP 2

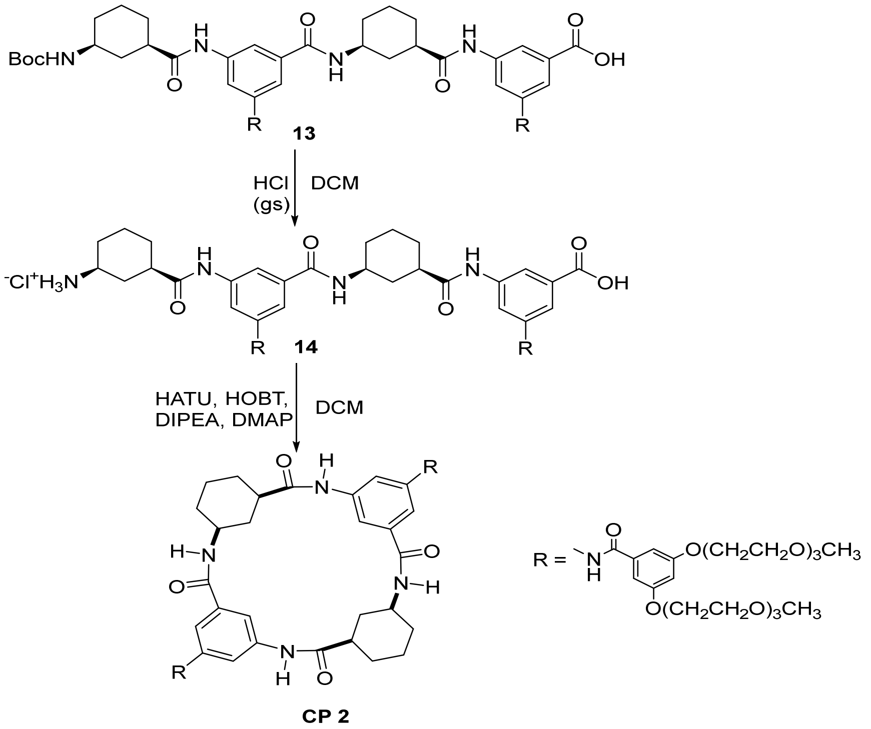

Scheme S2-4 The synthesis of **CP 2**

CP 2: Compound **13** (51 mg, 0.034 mmol) was dissolved in CH_2Cl_2 (10 mL). HCl (g) was introduced into the solution and stirred for 20-30 min. The reaction was monitored by TLC (CH_2Cl_2:MeOH:EA=10:1:1), and after the reaction was completed the solvent was removed under reduced pressure to get **14** as a light yellow solid and the product was dissolved in anhydrous CH_2Cl_2 (10 mL, 4mmol/L). To the solution, HATU (0.0129 g, 0.034 mmol), HOBt (0.0046 g, 0.034 mmol), DMAP (0.0021 g, 0.017 mmol) and DIPEA (0.03 mL, 0.170 mmol) were added at -10-0 ºC for 1 h. Then the mixture was warmed up to room temperature slowly and stirred for 48 h at room temperature. After stirring the resulting solution until completion of the reaction, 1M HCl (aq) was poured into the mixture and the product was extracted with CH_2Cl_2. The combined organic layers were washed with 3N $NaHCO_3$ (aq), water and brine. Then the organic phase was dried over anhydrous Na_2SO_4 and concentrated in vacuum, and the mixture was purified by column chromatography ($CHCl_3$:MeOH=50:1, TLC by CH_2Cl_2:MeOH:EA=10:1:1) and get CP **2** (0.0314 g, 66.6%) as a white foam solid.

^{1}H NMR (600 MHz, DMSO-d_6) δ 10.28 (s, 2H), 10.09 (m, 2H), 8.35 (s, 2H), 8.27 (d, J = 10.2 Hz, 2H), 7.79 (s, 2H), 7.72 (s, 2H), 7.14 (d, J = 14.1 Hz, 4H), 6.74 (s, 2H),

4.16 (s, 8H), 3.86 (s, 2H), 3.75 (s, 8H), 3.58 (s, 8H), 3.55 – 3.47 (m, 16H), 3.41 (d, *J* = 3.1 Hz, 8H), 3.27 – 3.15 (m, 12H), 2.52 (s, 1H), 1.99 (s, 2H), 1.84 (s, 5H), 1.78 (s, 1H), 1.52 (t, *J* = 10.8 Hz, 2H), 1.35 (dd, *J* = 12.0, 11.4 Hz, 6H), 1.22 (d, *J* = 18.0 Hz, 1H).

^{13}C NMR (151 MHz, DMSO-d$_6$) δ 173.96, 166.33, 165.27, 160.02, 139.82, 139.48, 136.91, 136.57, 115.22, 114.62, 106.73, 104.67, 71.72, 70.41, 70.27, 70.06, 69.33, 67.94, 58.48, 48.23, 44.45, 35.50, 32.10, 28.85, 24.58.

ESI-HRMS: $C_{70}H_{98}N_6NaO_{22}$ $[M+Na]^+$ calculated: 1397.6626, found: 1397.5540.

2.5 Synthesis methods of CP 3

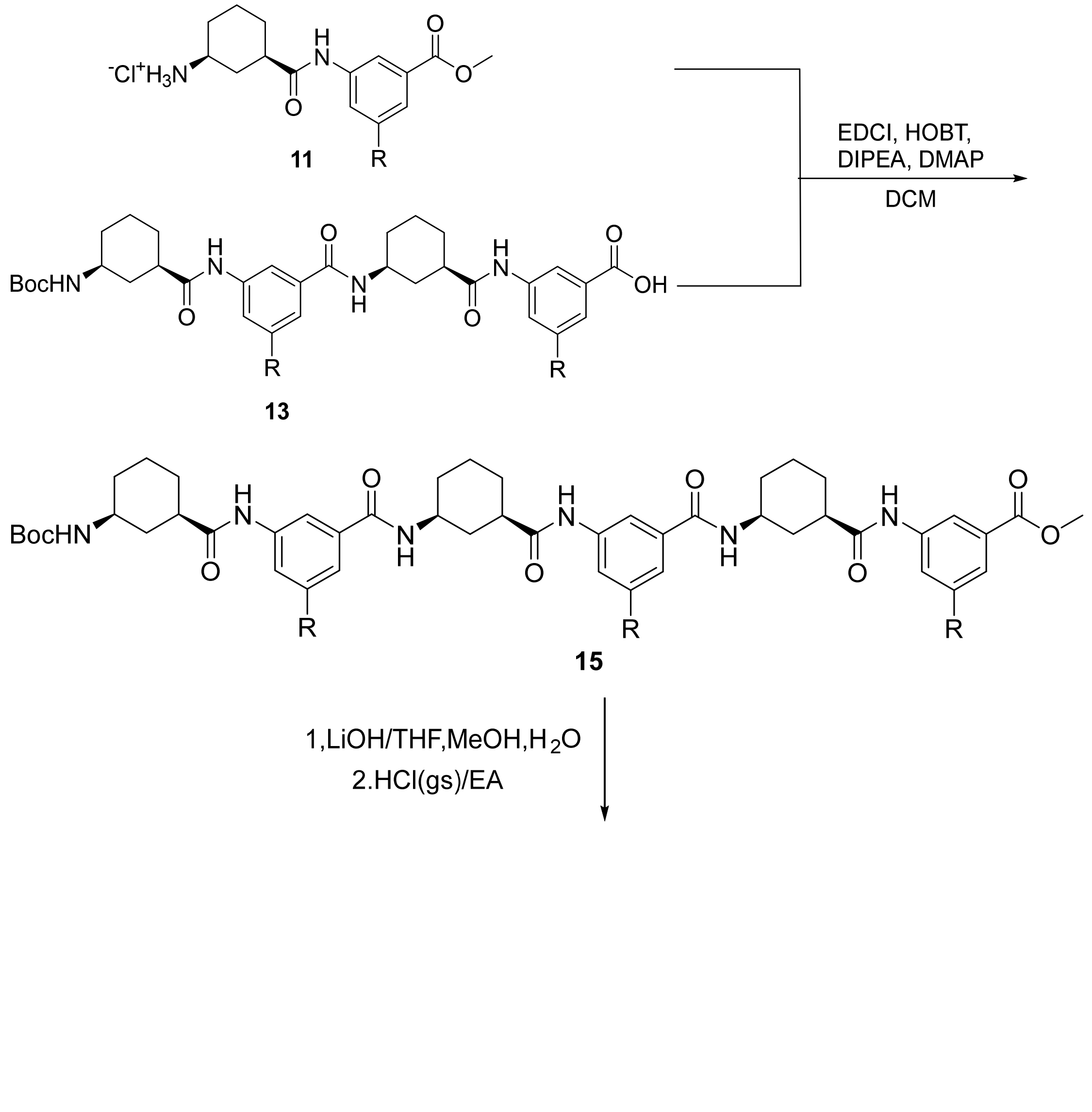

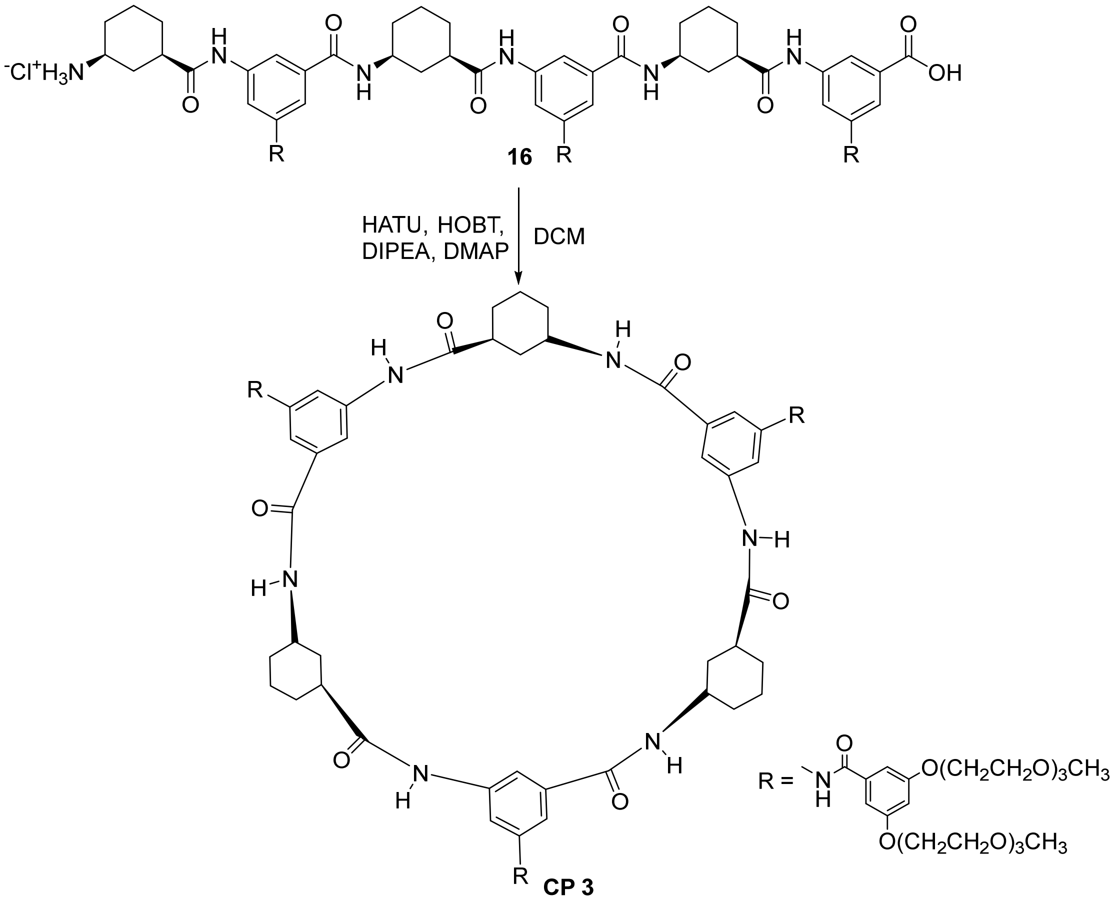

Scheme S2-5 The synthesis of CP **3**.

The synthesis procedures:

Hexapeptide 15：To a solution of **13** (0.146 g, 0.098 mmol), EDCI (0.038 g, 0.196 mmol), HOBt (0.027 g, 0.196 mmol) and DMAP (0.012 g, 0.098 mmol) in CH_2Cl_2 (5 mL) at -10-0 ºC for 1 h, were added **11** (0.071 g, 0.098 mmol) dissolved in CH_2Cl_2 (5 mL) and DIPEA (0.33 mL, 1.96 mmol). Then the mixture was warmed up to room temperature slowly and stirred for 48 h at room temperature. After stirring the resulting solution until completion of the reaction, 1M HCl (aq) was poured into the mixture and the product was extracted with CH_2Cl_2. The combined organic layers were washed with 3N $NaHCO_3$ (aq), water and brine. Then the organic phase was dried over anhydrous Na_2SO_4 and concentrated in vacuum, and the mixture was purified by column chromatography ($CHCl_3$:MeOH=50:1, TLC by $CHCl_3$:MeOH=15:1) and get **15** (0.159 g, 65.8%) as a colorless oil.

^{1}H NMR (600 MHz, DMSO-d_6) δ 10.40 – 10.31 (m, 2H), 10.27 (d, *J* = 3.2 Hz, 1H), 10.22 (d, *J* = 13.6 Hz, 1H), 10.17 (s, 1H), 10.10 (s, 1H), 10.04 (s, 1H), 8.44 (t, *J* = 13.4 Hz, 2H), 8.35 (t, *J* = 7.2

Hz, 1H), 8.28 (t, *J* = 10.3 Hz, 1H), 8.08 (d, *J* = 4.6 Hz, 2H), 8.04 (d, *J* = 9.7 Hz, 2H), 7.83 (d, *J* = 7.9 Hz, 1H), 7.78 (d, *J* = 6.7 Hz, 1H), 7.72 (d, *J* = 7.9 Hz, 1H), 7.16 (t, *J* = 7.4 Hz, 6H), 6.84 – 6.72 (m, 3H), 4.16 (d, *J* = 2.9 Hz, 12H), 3.90 – 3.84 (m, 6H), 3.76 (d, *J* = 3.0 Hz, 12H), 3.60 (dd, *J* = 9.0, 4.0 Hz, 12H), 3.57 – 3.49 (m, 24H), 3.27 – 3.19 (m, 17H), 2.53 (s, 1H), 2.49 – 2.38 (m, 2H), 2.05 – 1.69 (m, 15H), 1.62 – 1.01 (m, 25H), 0.86 (ddd, *J* = 7.8, 6.0, 7.8 Hz, 2H).

^{13}C NMR (151 MHz, DMSO-d$_6$) δ 174.20, 174.06, 173.97, 168.88, 166.52, 166.38, 165.54, 165.36, 160.02, 140.34, 140.32, 140.08, 139.78, 139.45, 136.89, 136.80, 136.51, 130.57, 116.34, 115.94, 115.65, 106.80, 106.72, 104.73, 78.00, 71.71, 70.40, 70.25, 70.05, 69.32, 67.97, 58.49, 58.48, 52.74, 49.06, 48.25, 47.46, 44.43, 44.37, 35.65, 32.35, 28.70, 24.54, 24.43, 23.16.

CP 3: Under a ice water condition, Compound **15** (0.162 g, 0.074mmol) was dissolved in THF (5 mL) and MeOH (10 mL) mixed solvent and Lithium Hydroxide Monohydrate aqueous solution (0.016 g/5 mL) was added, keep this condition stirring for 0.5 hour and then moved to room temperature stirred overnight. The reaction was monitored by TLC ($CHCl_3$:MeOH=12:1), and after the reaction was completed the solvent was removed under reduced pressure. The aqueous phase was then acidified with 1M HCl (aq) to pH 2, extracted with CH_2Cl_2, then the organic phase was dried over anhydrous Na_2SO_4 and concentrated in vacuumto get a light yellow oil (0.150 g, 93.1%). Then the light yellow oil was dissolved in CH_2Cl_2 (10 mL). HCl (g) is introduced into the solution and stirred for 20-30 min. Testing of raw materials reacted complete by TLC ($CHCl_3$:MeOH=9:1), and the solvent was removed under reduced pressure to get **16** as a light yellow solid and the product **16** (0.155 g, 0.074 mmol) was then dissolved in anhydrous CH_2Cl_2 (19 mL, 4mmol/L). To the solution, HATU (0.028 g, 0.074 mmol), HOBt (0.010 g, 0.074 mmol), DMAP (0.005, 0.037 mmol) and DIPEA (0.12 mL, 0.74 mmol) were added at -10-0 ºC for 1 h. Then the mixture was warmed up to room temperature slowly and stirred for 5 days at room temperature. After stirring the resulting solution until completion of the reaction, 1M HCl (aq) was poured into the mixture and the product was extracted with CH_2Cl_2. The combined organic layers were washed with 3N $NaHCO_3$ (aq), water and brine. Then the organic phase was dried over anhydrous Na_2SO_4 and concentrated in vacuum, and the mixture was purified by

column chromatography ($CHCl_3$:MeOH=100:1, TLC by $CHCl_3$:MeOH=13:1) and get **CP 3** (0.063 g, 41.1%) as a white foam solid.

^{1}H NMR (600 MHz, DMSO-d_6) δ 10.28 (s, 3H), 10.19 – 10.03 (m, 3H), 8.35 (s, 2H), 8.26 (m, 3H), 8.15 – 8.07 (m, 1H), 8.02 (d, *J* = 12.2 Hz, 1H), 7.86 – 7.75 (m, 3H), 7.75 – 7.69 (m, 2H), 7.23 – 7.10 (m, 6H), 6.74 (t, *J* = 9.0 Hz, 3H), 4.14 (dd, *J* = 4.2, 6.0 Hz, 12H), 3.93 – 3.82 (m, 3H), 3.80 – 3.71 (m, 12H), 3.59 (dt, *J* = 3.6, 9.0 Hz, 12H), 3.53 (ddd, *J* = 4.8, 5.4, 3.6 Hz, 24H), 3.45 – 3.39 (m, 12H), 3.24 – 3.19 (m, 18H), 2.53 (d, *J* = 3.9 Hz, 2H), 2.06 – 1.96 (m, 3H), 1.83 (m, 10H), 1.55 (d, *J* = 11.2 Hz, 2H), 1.44 – 1.27 (m, 10H).

^{13}C NMR (151 MHz, DMSO-d_6) δ 204.33, 198.40, 189.24, 173.97, 171.35, 171.26, 167.68, 167.41, 166.34, 165.27, 160.02, 145.33, 139.84, 136.92, 133.74, 106.78, 106.78, 106.75, 106.74, 106.73, 104.69, 100.00, 71.74, 71.72, 70.42, 70.41, 70.29, 70.27, 70.08, 70.06, 69.34, 67.94, 58.52, 58.48, 22.58.

ESI-HRMS: $C_{105}H_{148}N_9NaO_{33}$ $[M+Na]^+$ calculated: 2086.3204, found: 2086.4247.

3. ^{1}H NMR and ^{13}C NMR spectra

The ^{1}H NMR (600 MHz, DMSO-d6) spectrum of **4**

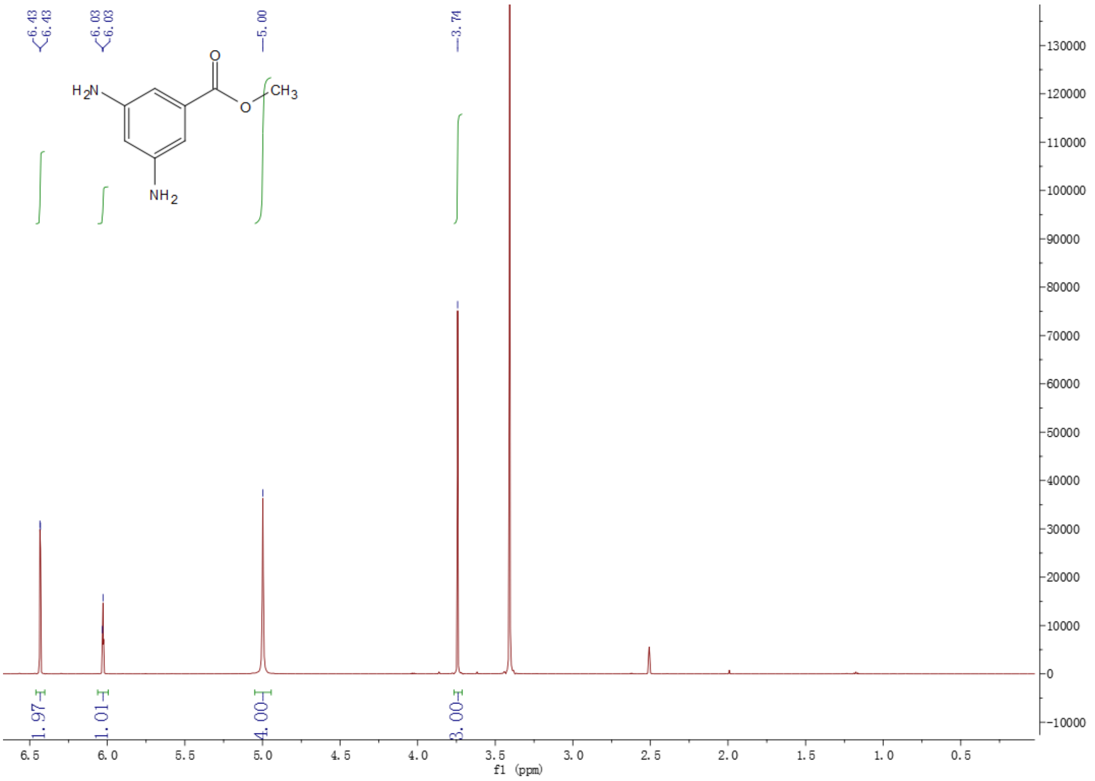

The ^{1}H NMR (600 MHz, $CDCl_3$) spectrum of **5**

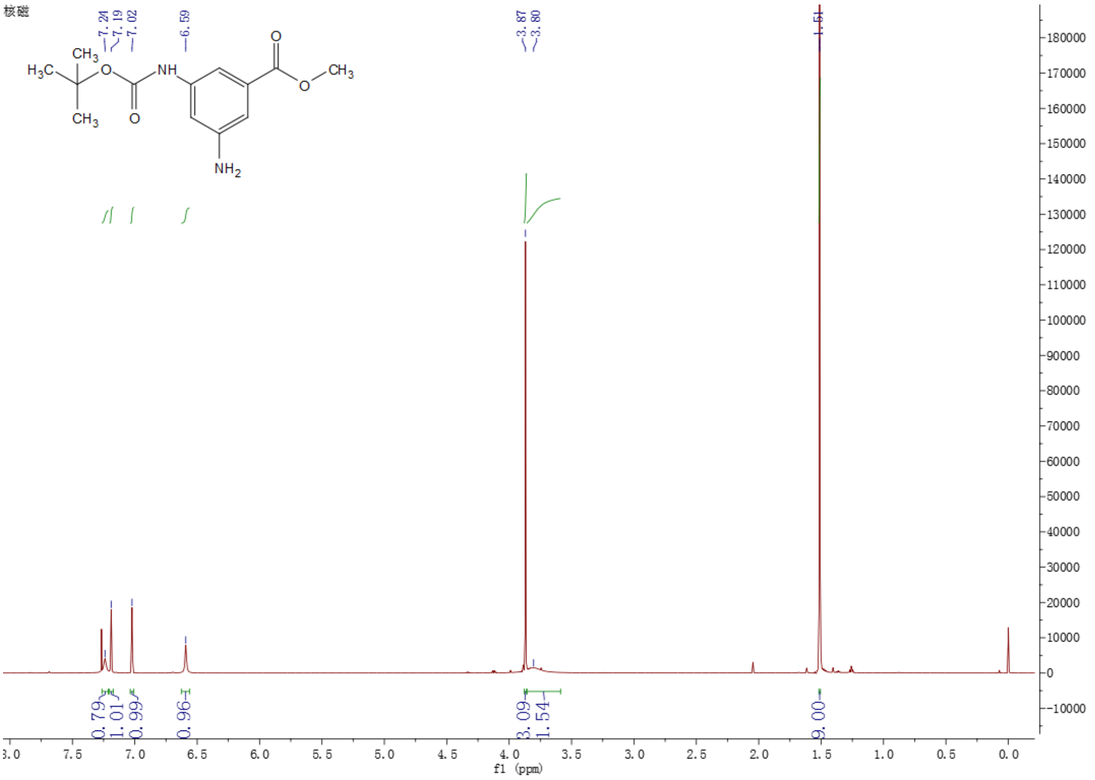

The ^{1}H NMR (600 MHz, $CDCl_3$) spectrum of compound **7**

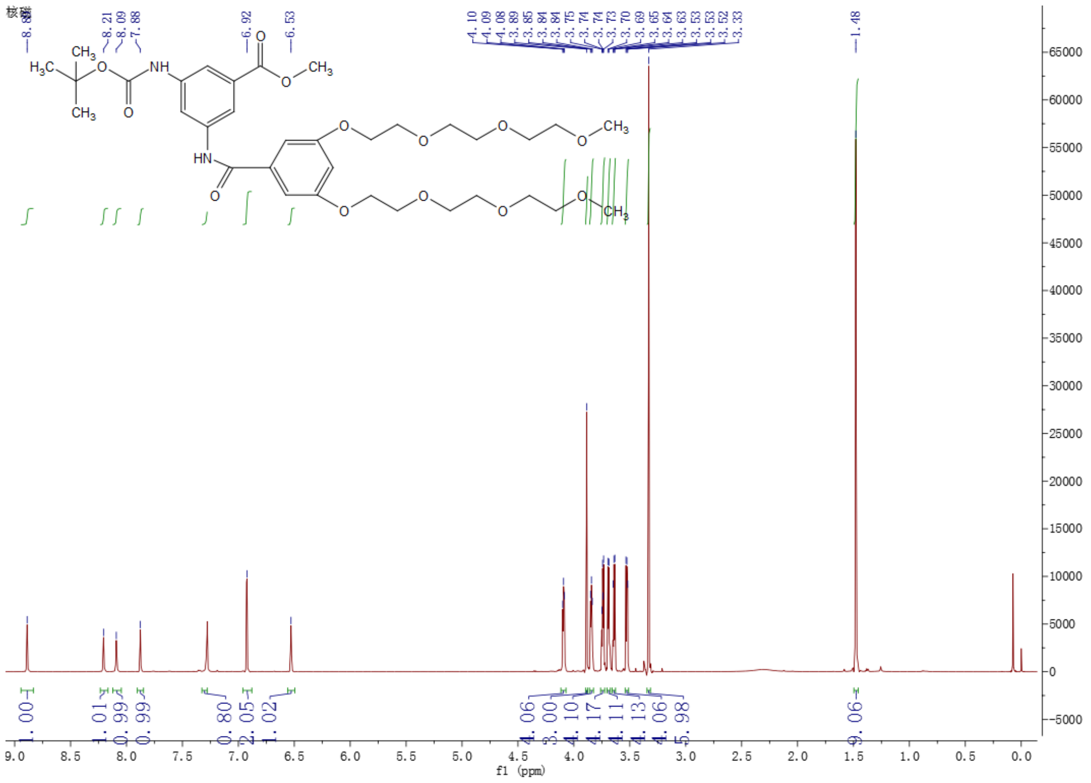

The ^{1}H NMR (600 MHz, $CDCl_3$) spectrum of compound **8**

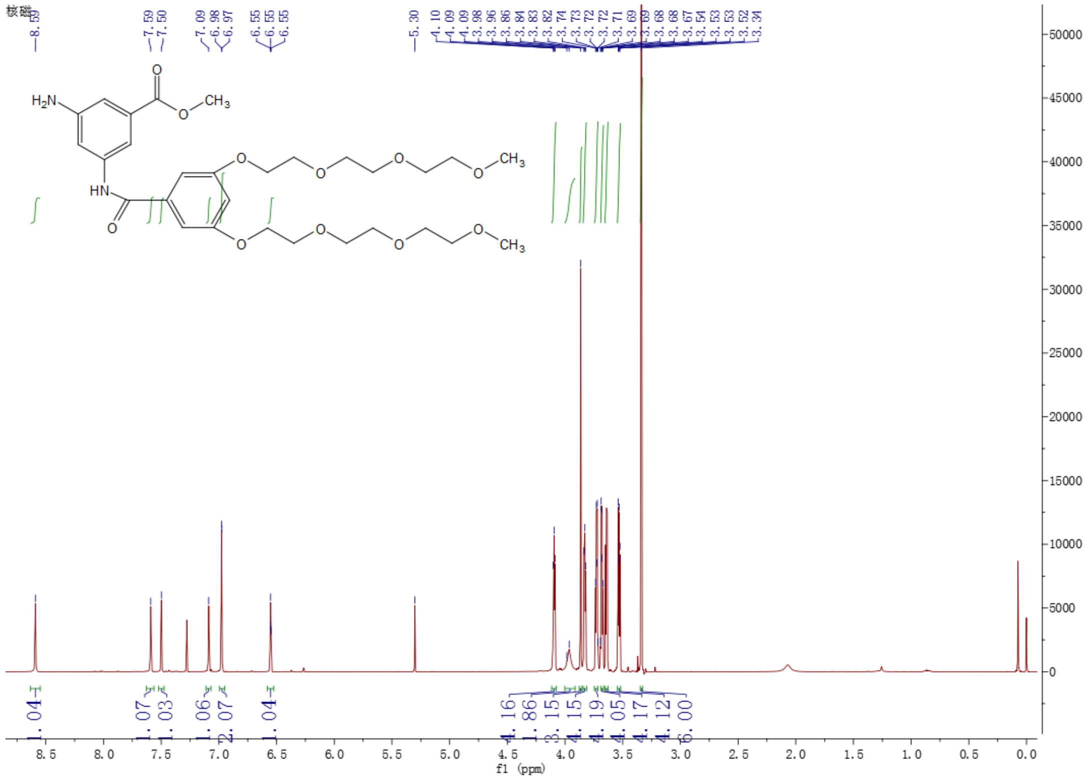

The ^{1}H NMR (600 MHz, $CDCl_3$) spectrum of **14**

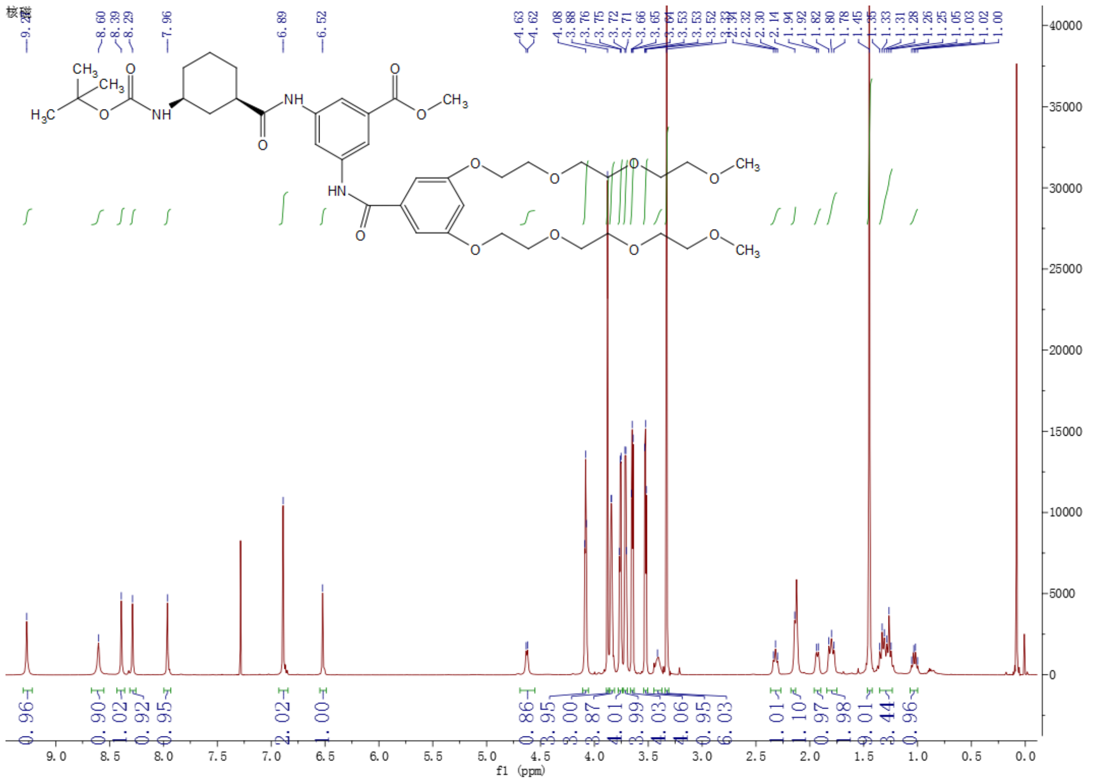

The ^{13}C NMR (151 MHz, $CDCl_3$) spectrum of **9**

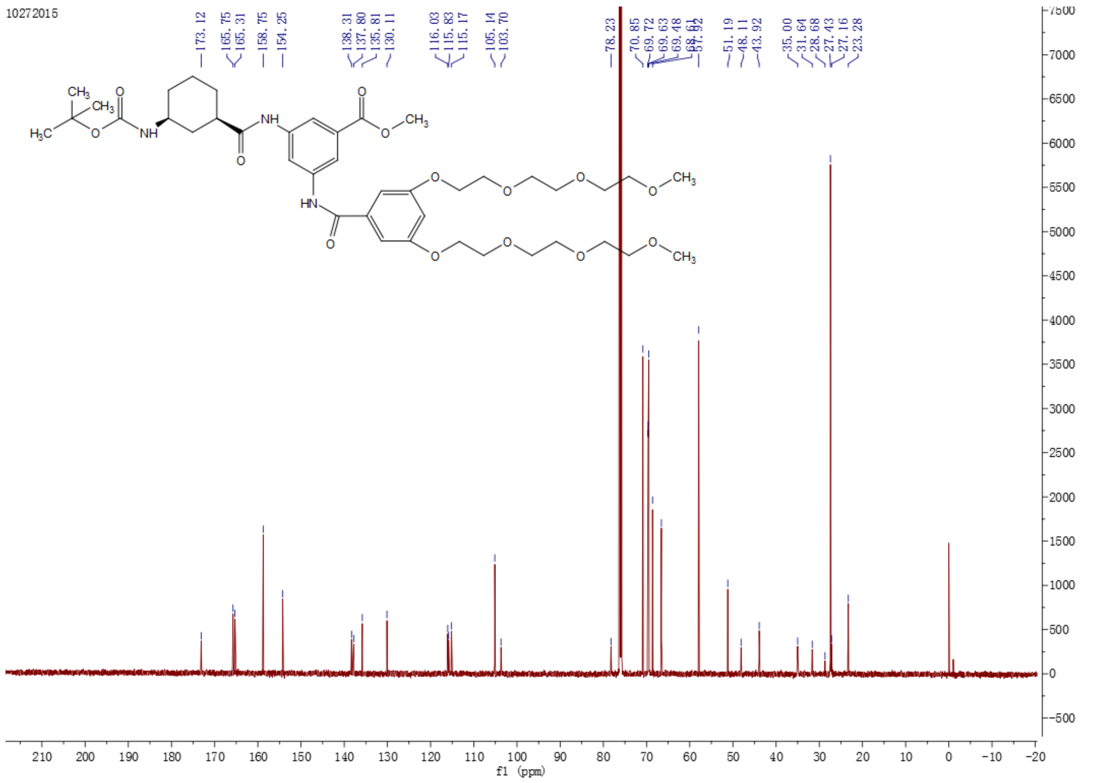

The ^{1}H NMR (600 MHz, DMSO-d$_6$) spectrum of **12**

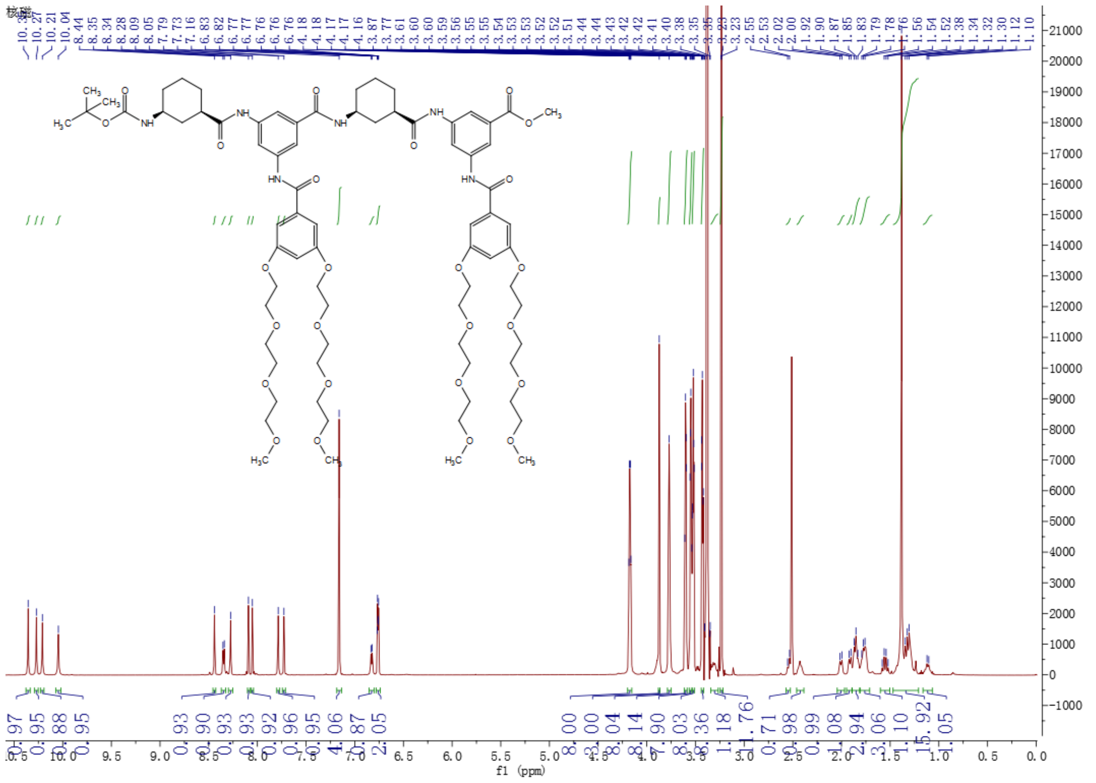

The ^{13}C NMR (151 MHz, DMSO-d$_6$) spectrum of **12**

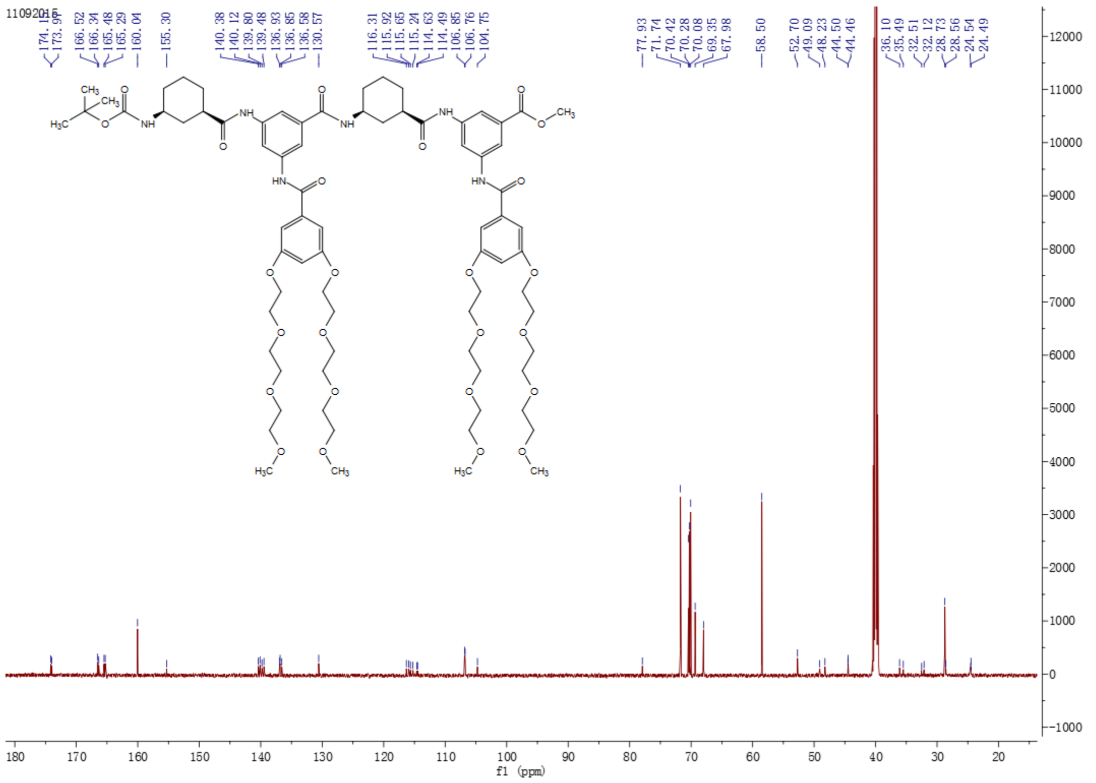

The ^{1}H NMR (600 MHz, DMSO-d_6) spectrum of CP **2**

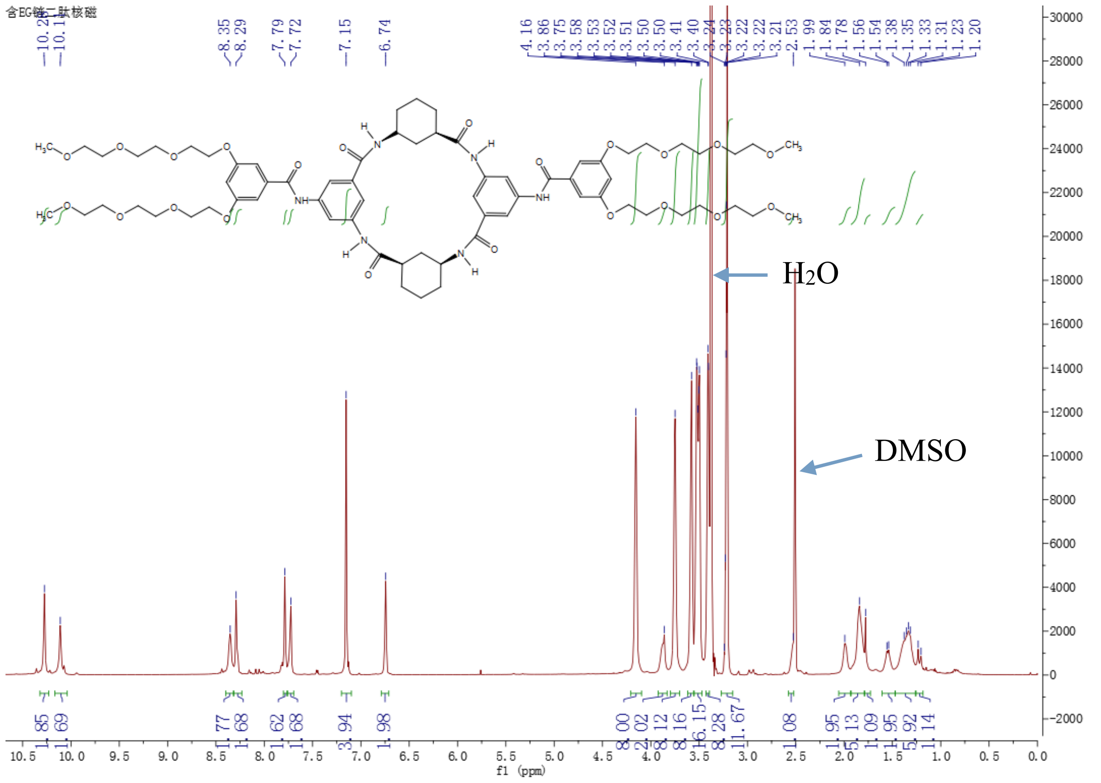

The ^{13}C NMR (151 MHz, DMSO-d_6) spectrum of CP **2**

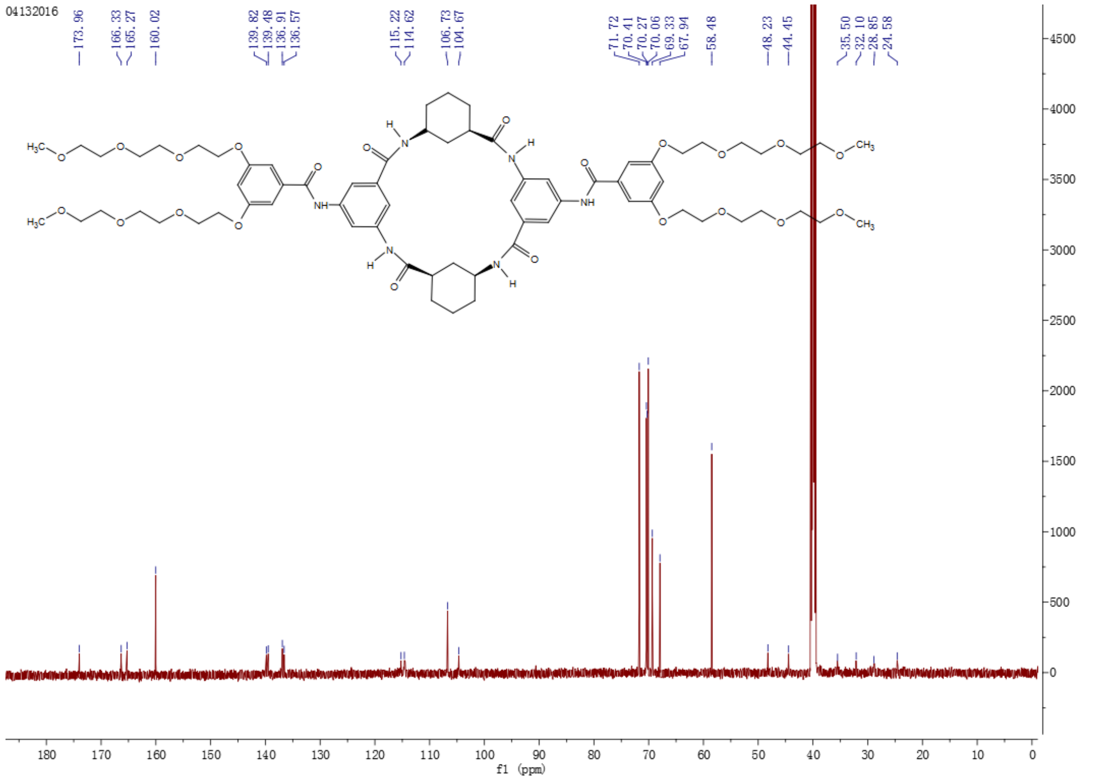

The ^{1}H NMR (600 MHz, DMSO-d_6) spectrum of **15**

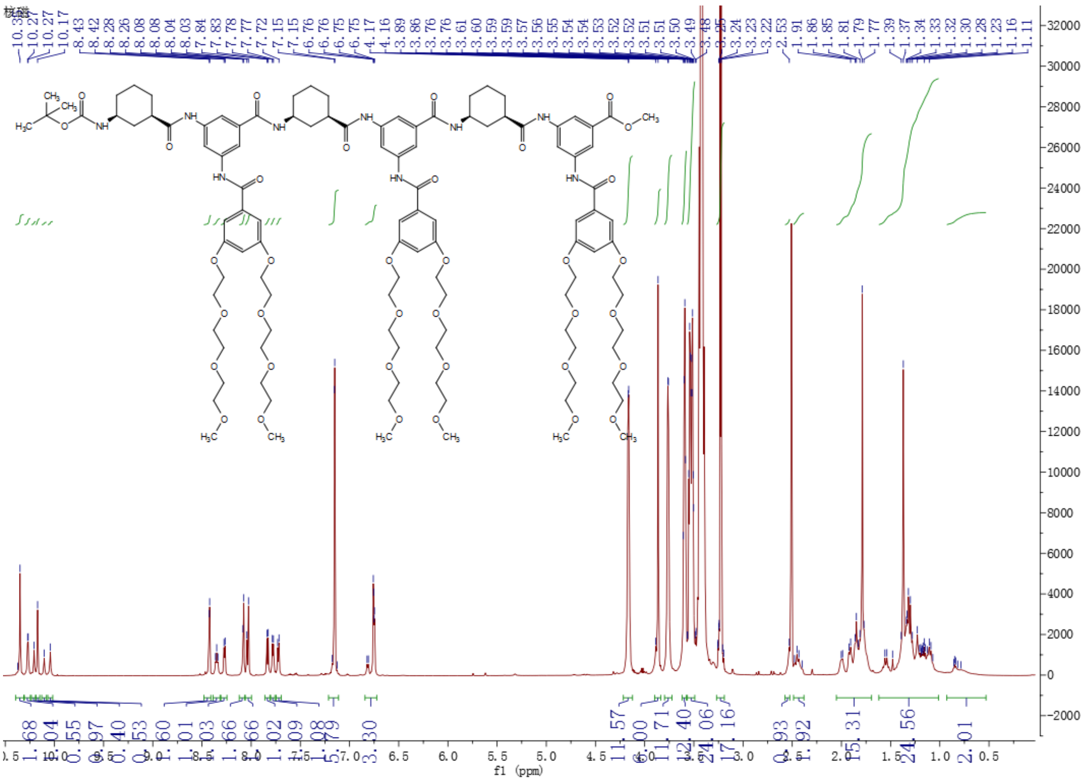

The ^{13}C NMR (151 MHz, DMSO-d_6) spectrum of **15**

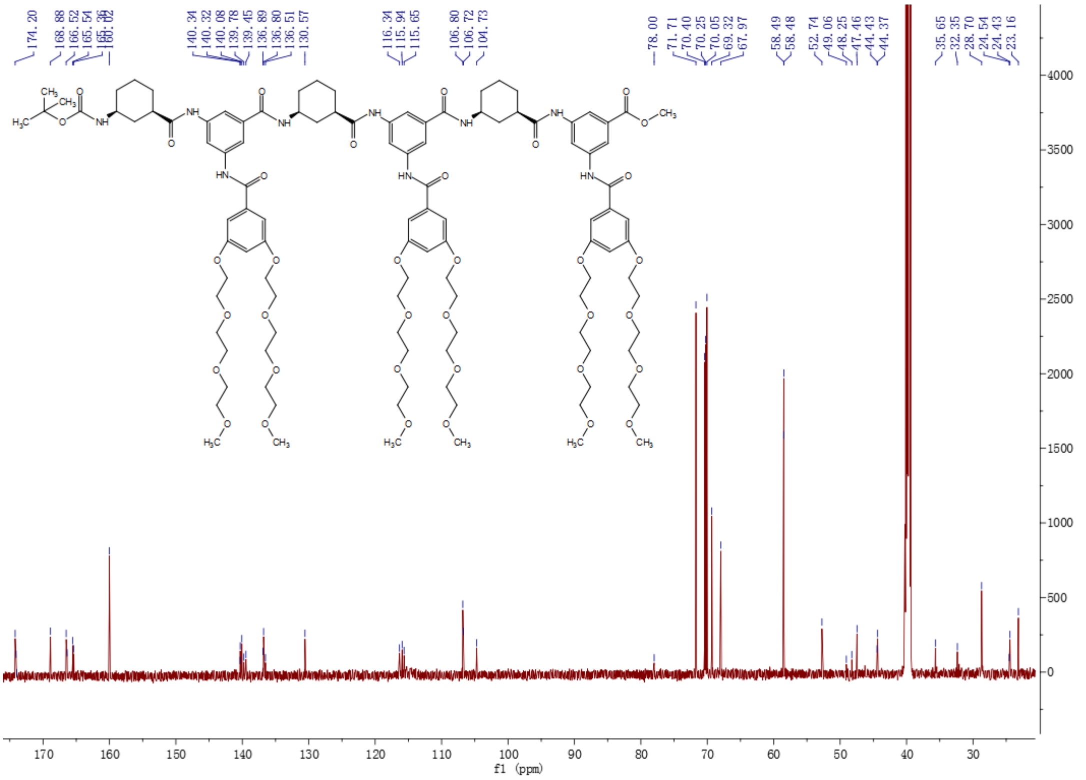

The ^{1}H NMR (600 MHz, DMSO-d_6) spectrum of CP **3**

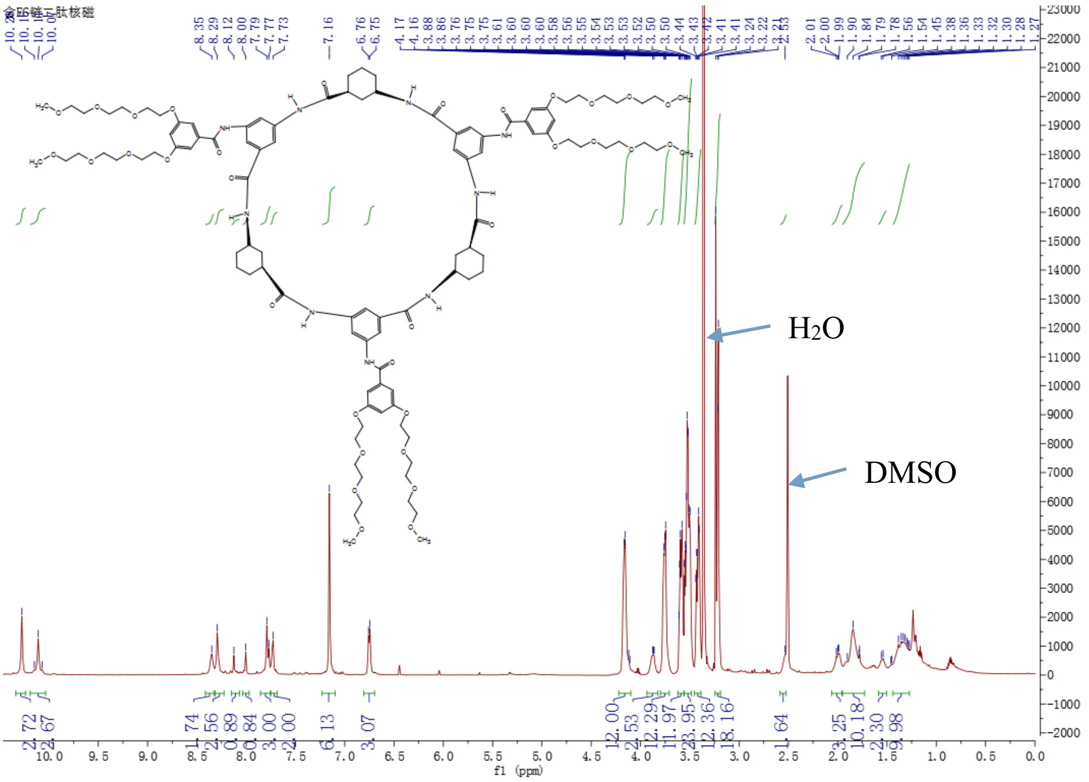

The ^{13}C NMR (151 MHz, DMSO-d_6) spectrum of CP **3**

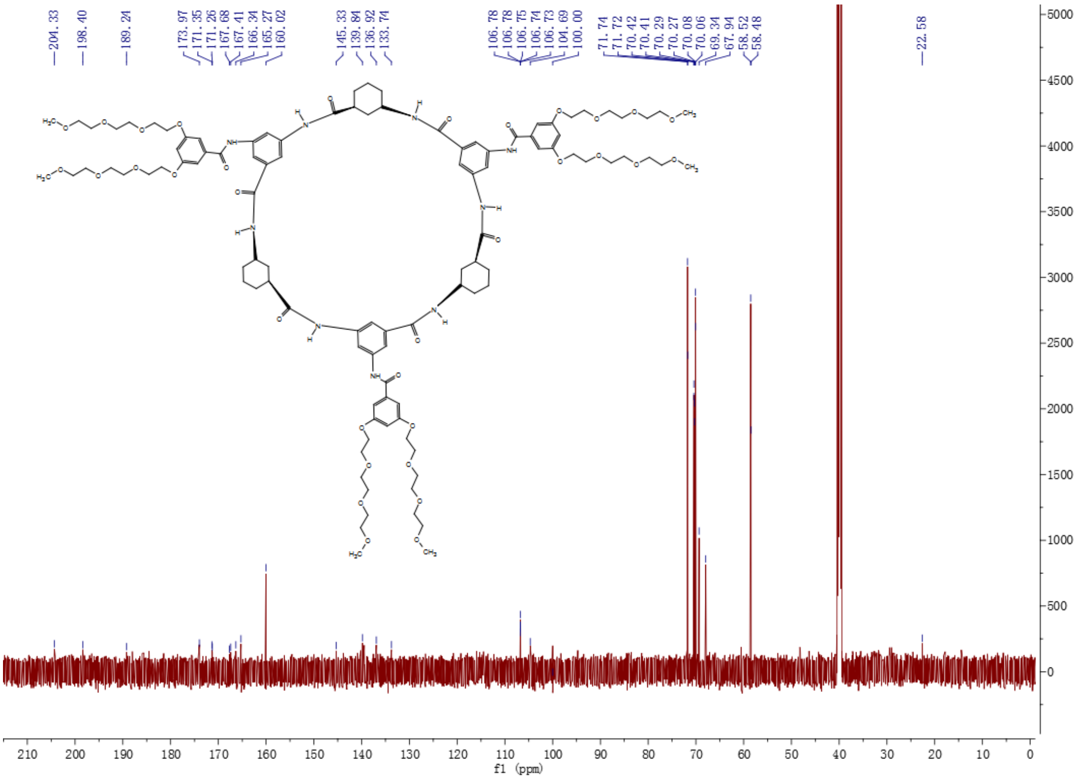

4.1The variable-concentration and variable-temperature ^{1}H NMR spectra of CP 2 and 3 [3-4]

The variable-concentration ^{1}H NMR spectra of CP **2** and **3**

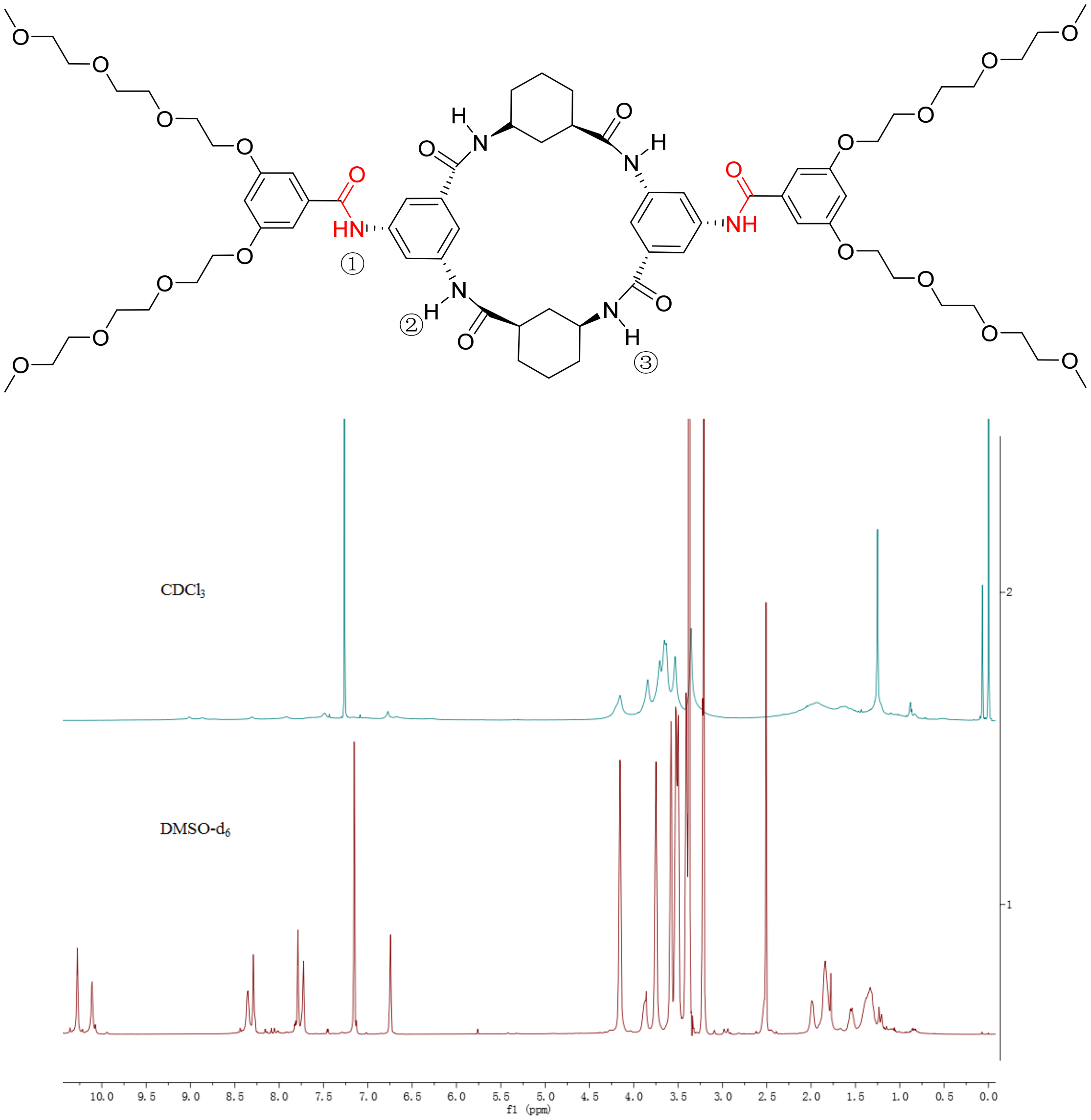

Figure S4-1 The ^{1}H NMR spectra of CP **2** in DMSO and $CDCl_3$.

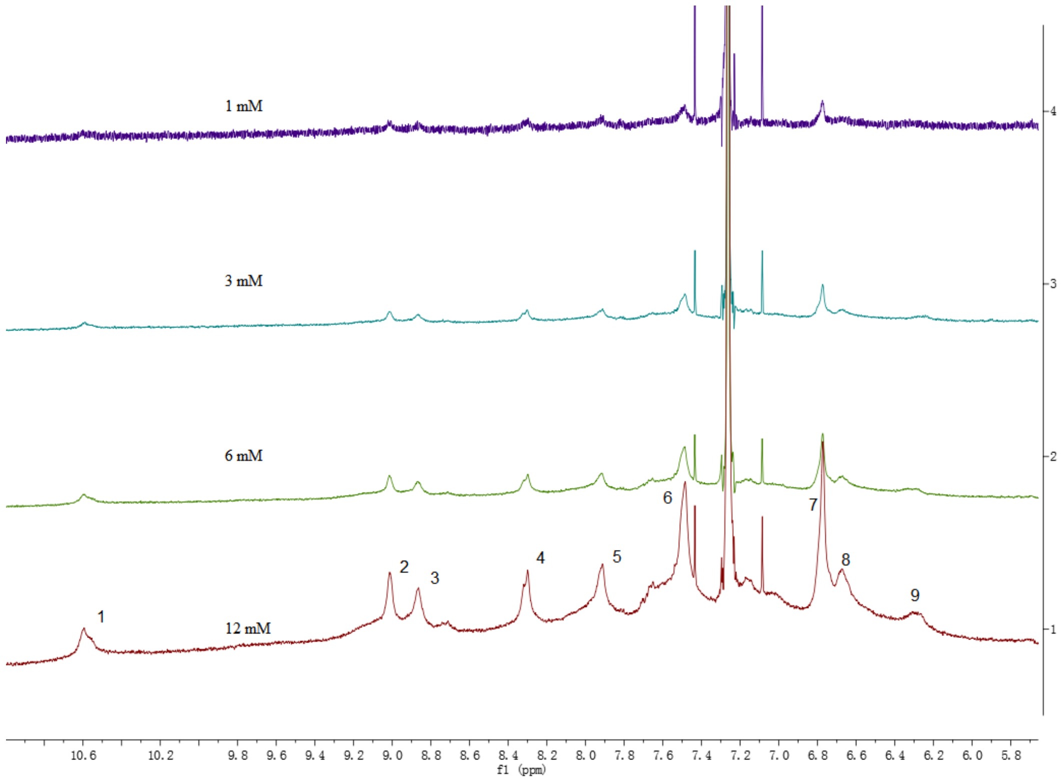

Figure S4-2 The variable-concentration ^{1}H NMR spectra of CP **2** in $CDCl_3$.

Table S4-1 The proton chemical shifts of CP **2** in different concentration in $CDCl_3$

Entry	Concentration (mM)			
	1	3	6	12
NH (1) (ppm)	10.6029	10.5985	10.5971	10.5944
NH (2) (ppm)	9.0175	9.0132	9.0114	9.0099
NH (3) (ppm)	8.8701	8.8679	8.8667	8.8650
4 (ppm)	8.2975	8.3001	8.3009	8.3036
5 (ppm)	7.9198	7.9106	7.9141	7.9149
6 (ppm)	7.4904	7.4889	7.4873	7.4857
7 (ppm)	6.7691	6.7707	6.7721	6.7726
8 (ppm)	6.6697	6.6739	6.6764	6.6785
9 (ppm)	6.2420	6.2427	6.2777	6.2905

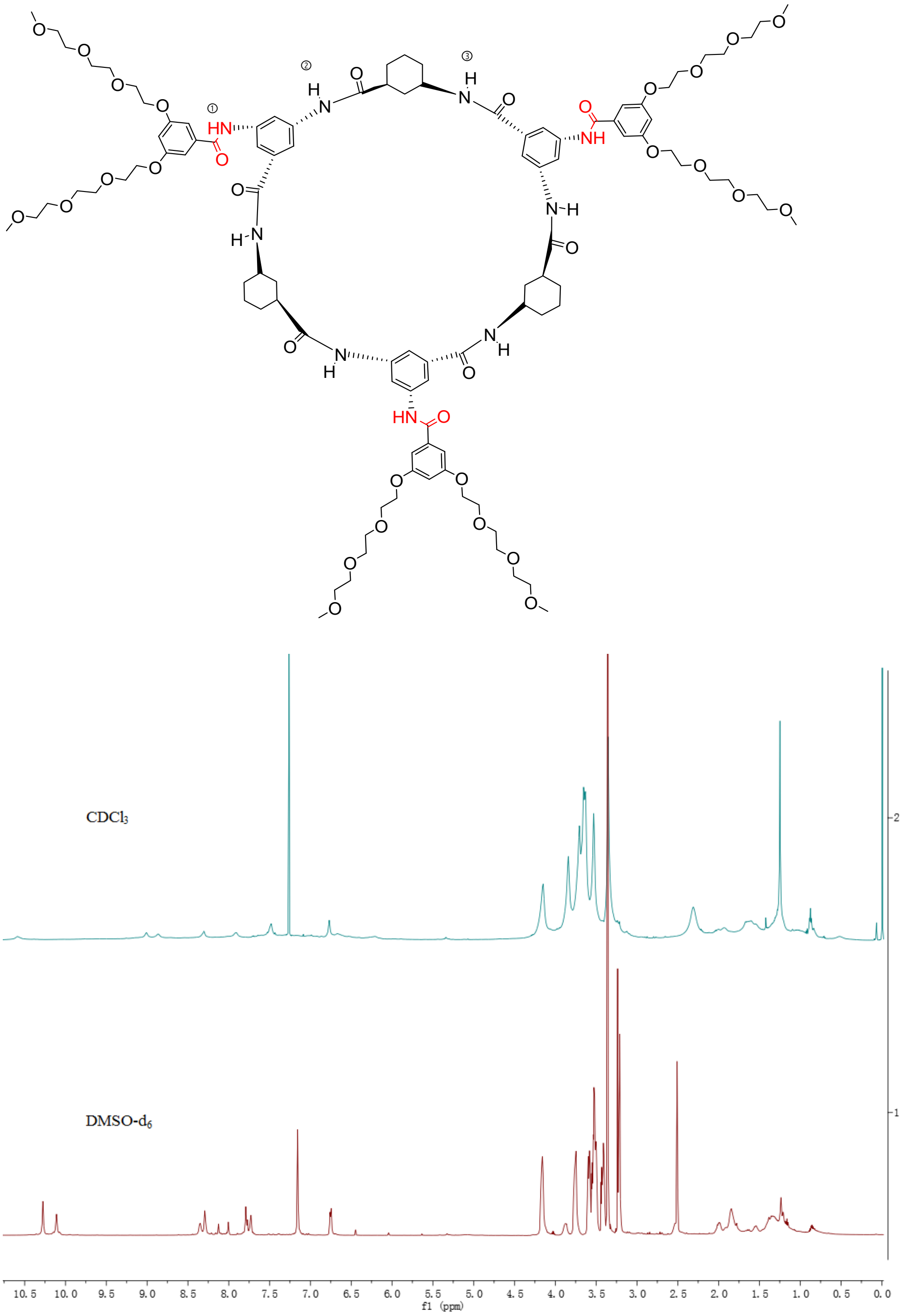

Figure S4-3 The ^{1}H NMR spectra of CP **3** in $CDCl_3$**.**

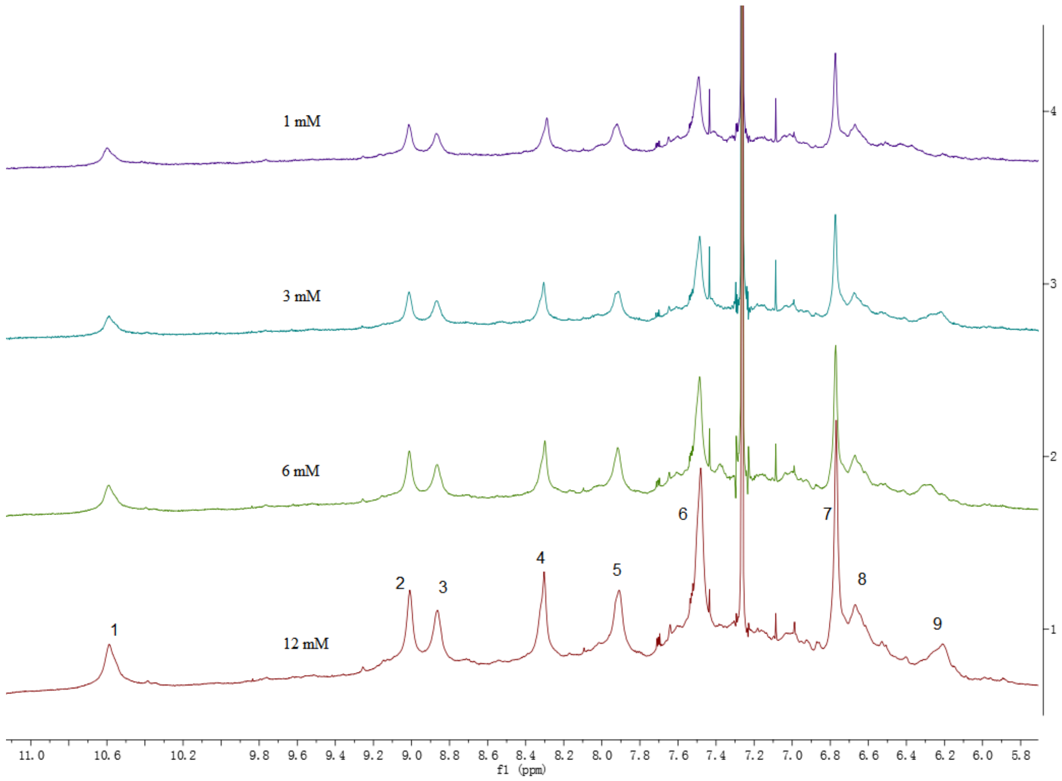

Figure S4-4 The variable-concentration ^{1}H NMR spectra of CP **3** in $CDCl_3$

Table S4-2 The proton chemical shifts of CP **3** in different concentration in $CDCl_3$

Entry	Concentration (mM)			
	1	3	6	12
NH (1) (ppm)	10.5997	10.5943	10.5906	10.5865
NH (2) (ppm)	9.0144	9.0122	9.0110	9.0073
NH (3) (ppm)	8.8704	8.8691	8.8663	8.8635
4 (ppm)	8.2879	8.3035	8.3048	8.3079
5 (ppm)	7.9272	7.9188	7.9177	7.9094
6 (ppm)	7.4909	7.4871	7.4859	7.4800
7 (ppm)	6.7691	6.7707	6.7721	6.7726
8 (ppm)	6.6707	6.6696	6.6687	6.6678
9 (ppm)	6.2126	6.2183	6.2875	6.2091

4.2 The variable-temperature ^{1}H NMR spectra of CP **2** and **3** in $CDCl_3$

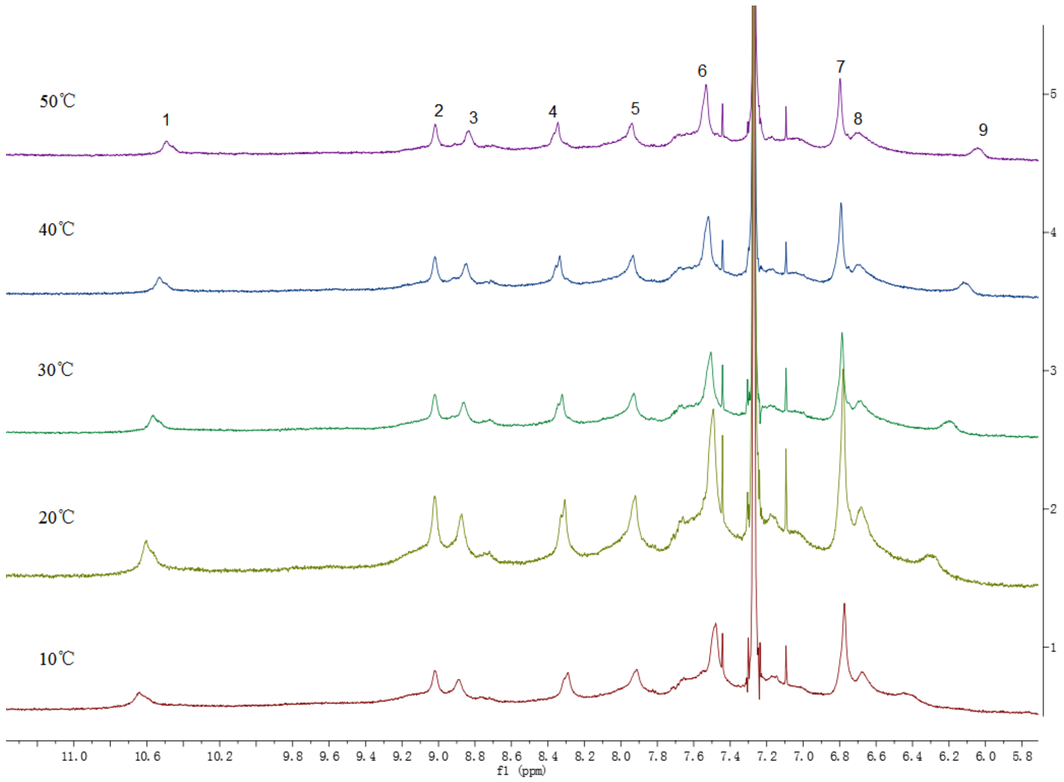

Figure S4-5 The variable-temperature ^{1}H NMR spectra of CP **2** in $CDCl_3$**.**

Table S4-3 The proton chemical shifts of CP **2** in different temperature in $CDCl_3$

Entry	Temperature (ºC)				
	10	20	30	40	50
**NH (1) (ppm)**	**10.6371**	**10.6068**	**10.5693**	**10.5247**	**10.4962**
NH (2) (ppm)	9.0227	9.0205	9.0187	9.0170	9.0152
NH (3) (ppm)	8.8847	8.8720	8.8609	8.8464	8.8371
4 (ppm)	8.2899	8.3058	8.3230	8.3356	8.3461
5 (ppm)	7.9124	7.9178	7.9266	7.9333	7.9405
6 (ppm)	7.4791	7.4929	7.5072	7.5194	7.5315
7 (ppm)	6.7721	6.7802	6.7878	6.7921	6.7978
8 (ppm)	6.6752	6.6809	6.6871	6.6952	6.6990
**9 (ppm)**	**6.4315**	**6.2932**	**6.1963**	**6.1108**	**6.0419**

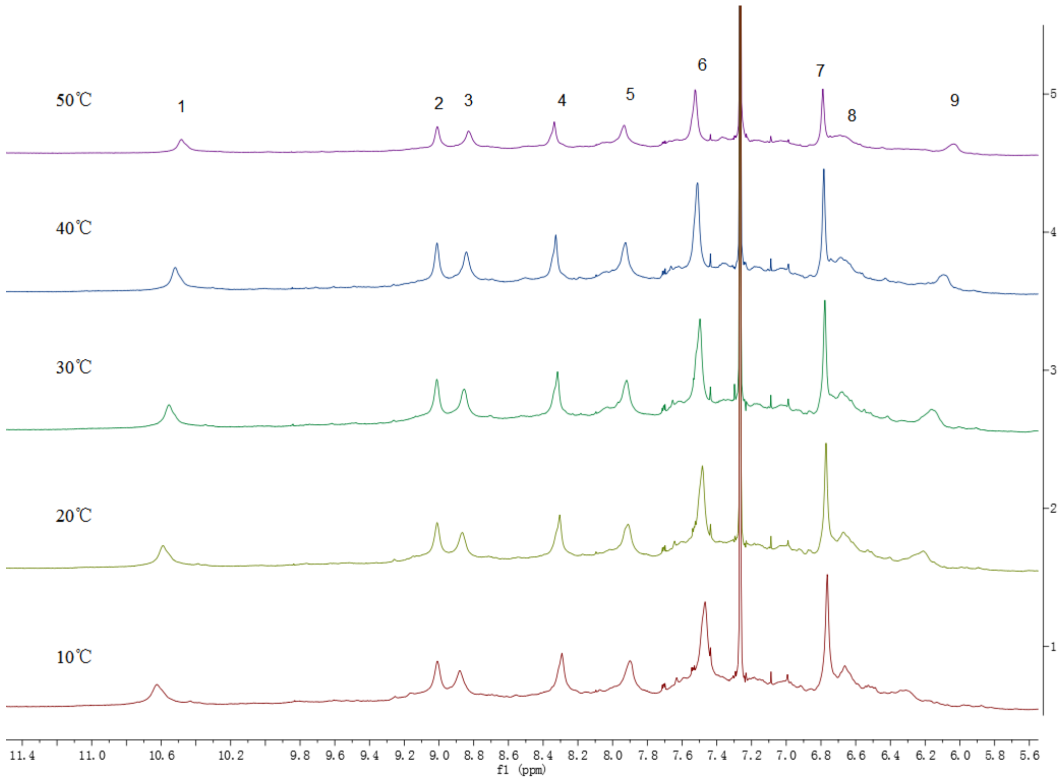

Figure S4-6 The variable-temperature ^{1}H NMR spectra of CP **3** in $CDCl_3$**.**

Table S4-4 The proton chemical shifts of CP **3** in different temperature in $CDCl_3$

Entry	Temperature (ºC)				
	10	20	30	40	50
**NH (1) (ppm)**	**10.6206**	**10.5888**	**10.5569**	**10.5176**	**10.4801**
NH (2) (ppm)	9.0129	9.0121	9.0113	9.0104	9.0086
NH (3) (ppm)	8.8799	8.8663	8.8568	8.8430	8.8284
4 (ppm)	8.2908	8.3051	8.3166	8.3247	8.3362
5 (ppm)	7.8910	7.9079	7.9187	7.9221	7.9329
6 (ppm)	7.4681	7.4823	7.4959	7.5101	7.5243
7 (ppm)	6.7646	6.7713	6.7754	6.7817	6.7904
8 (ppm)	6.6570	6.6652	6.6678	6.6720	6.6828
**9 (ppm)**	**6.3077**	**6.2084**	**6.1567**	**6.0899**	**6.0341**

5. HSQC and NOESY spectra of 2 and 3 in $CDCl_3$

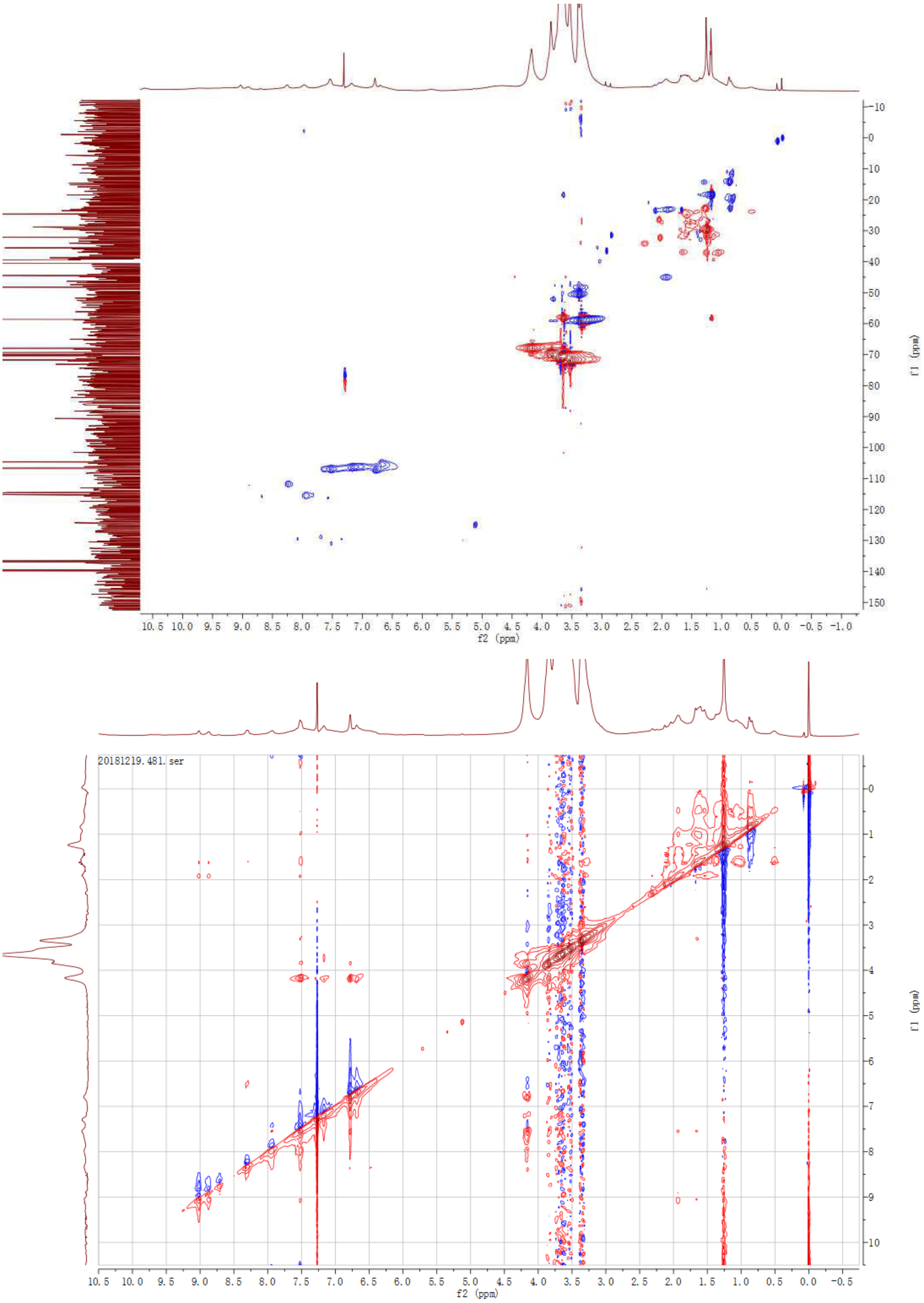

Figure S5-1. HSQC and NOESY spectra of **2** in $CDCl_3$.

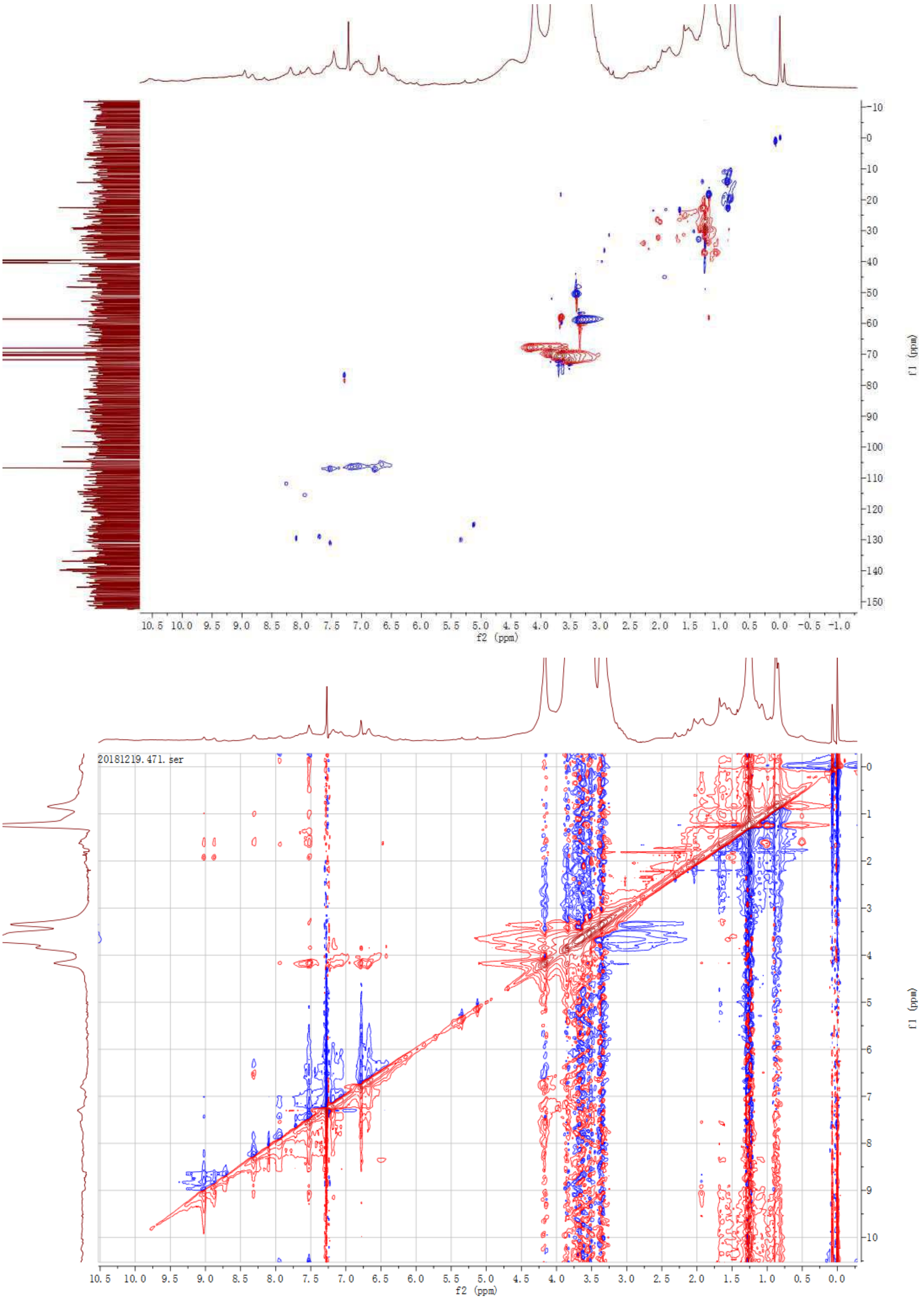

Figure S5-2. HSQC and NOESY spectra of **3** in $CDCl_3$.

6. The FT-IR spectra of CP 2 and 3 [5-8]

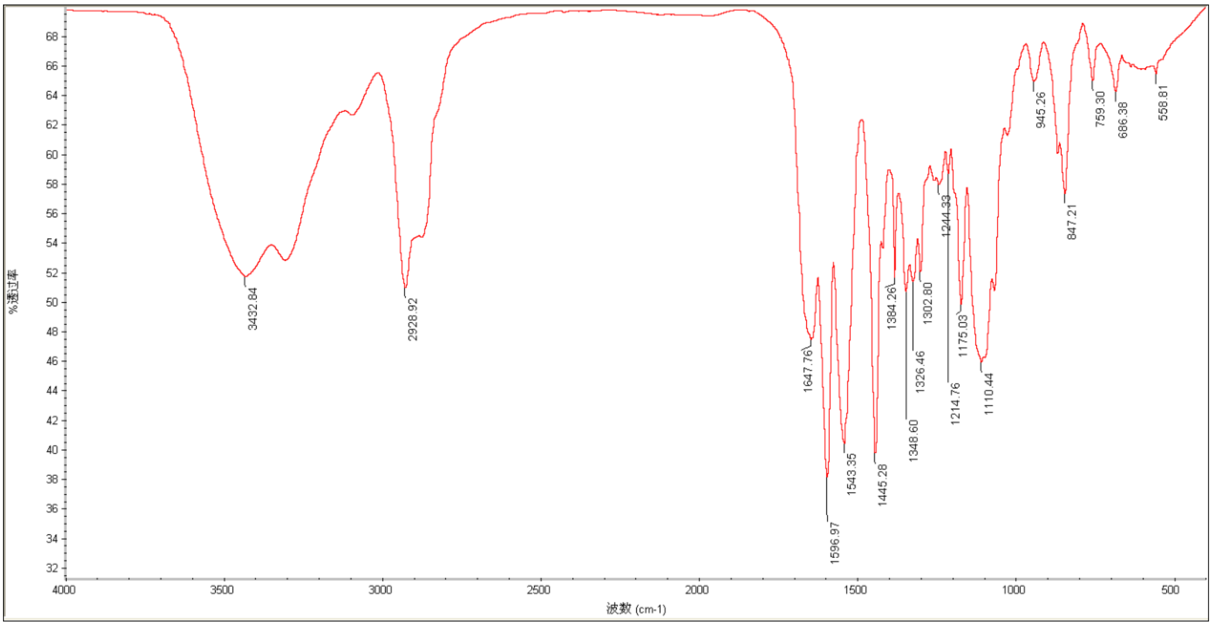

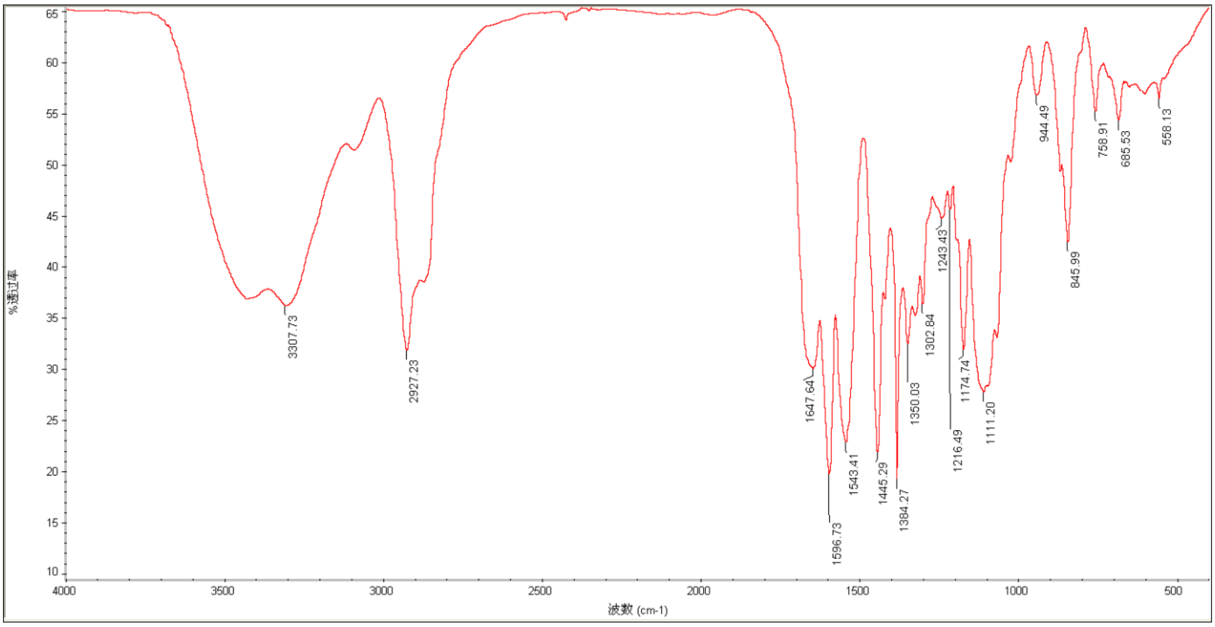

Figure S6 The FT-IR spectra (KBr) of CP **2** (upper) and CP **3** (down)

7. MALDI-TOF Results

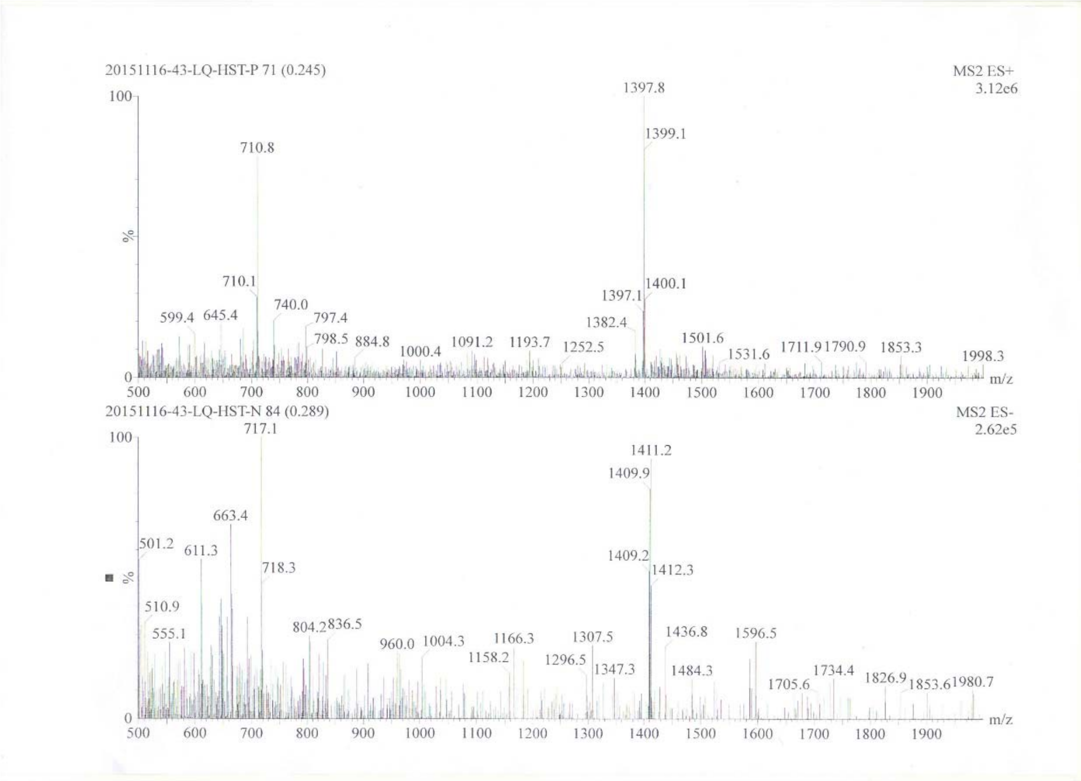

Figure S7-1 The HRMS spectrum of CP **2**

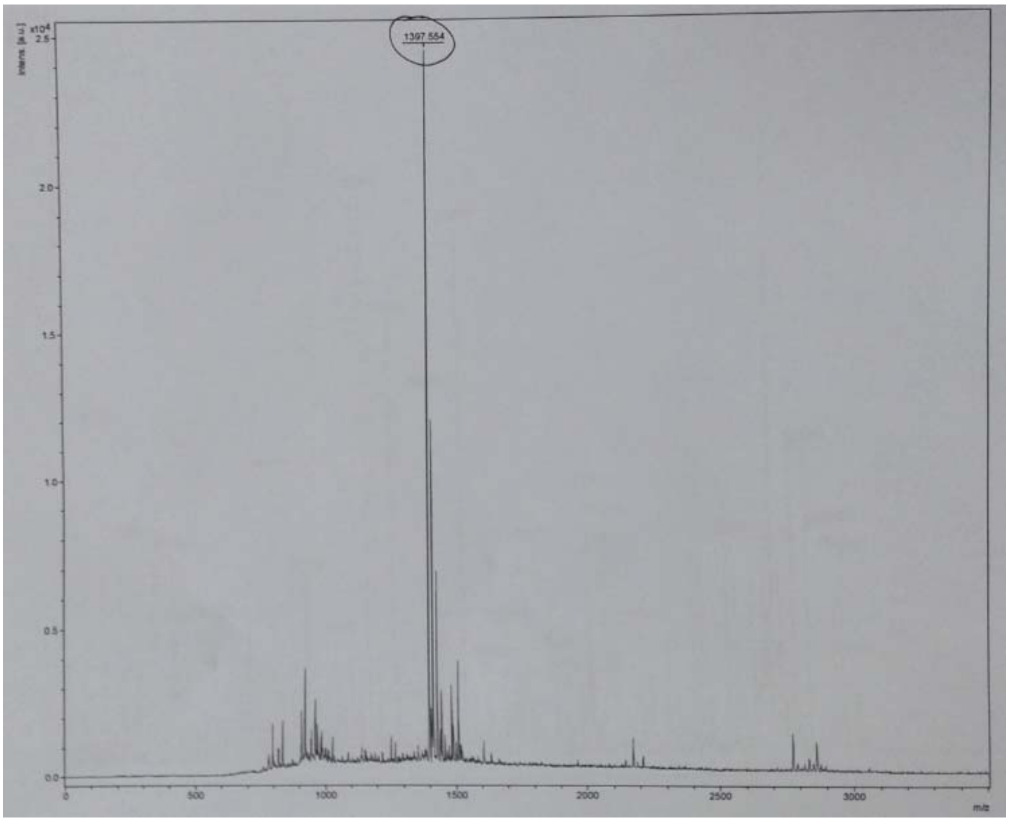

Figure S7-2 The MALDI-TOF-MS spectrum of CP **2**.

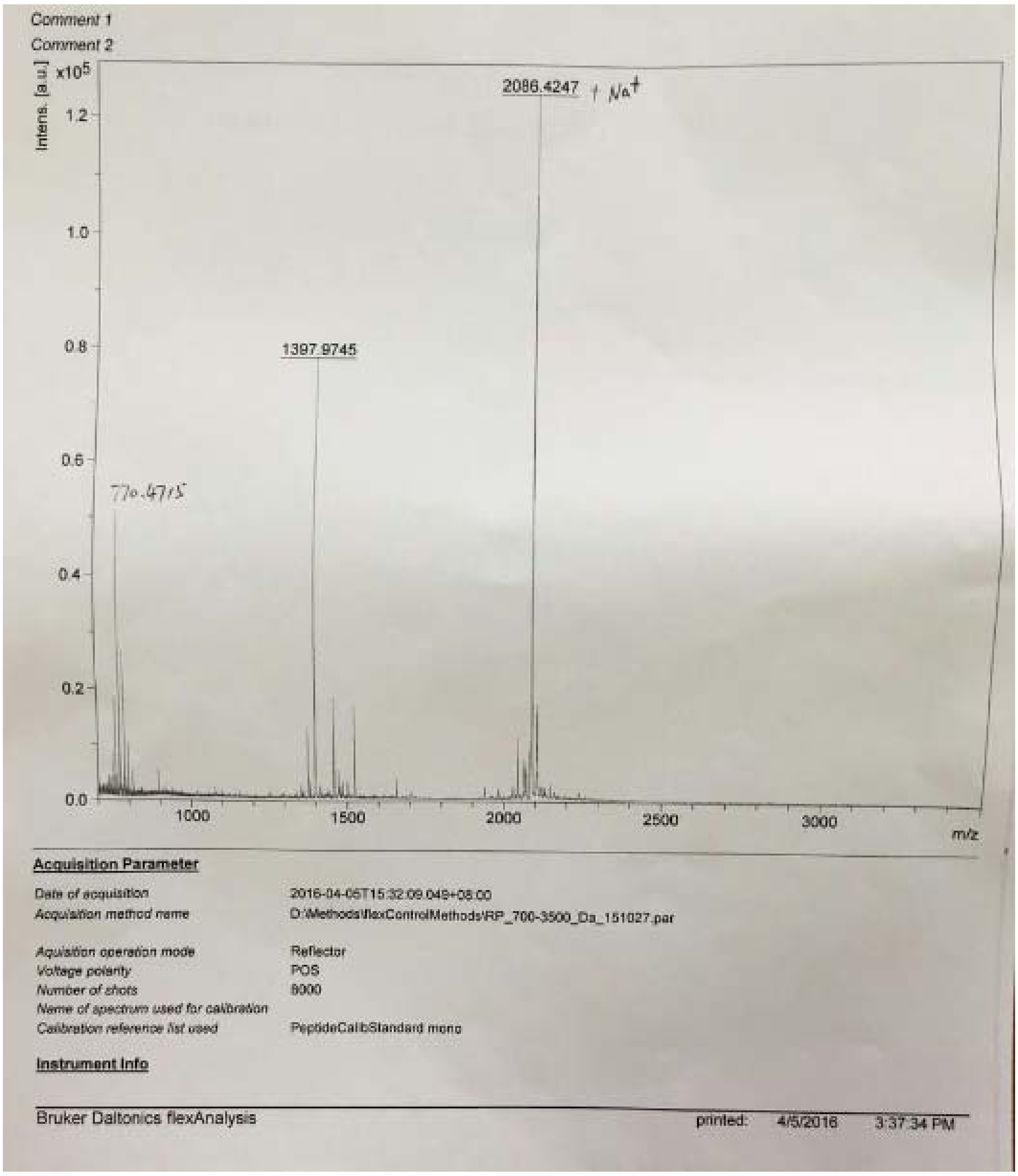

Figure S7-3 The MALDI-TOF-MS spectrum of CP **3**

8. The UV-vis spectra of CP 2 and CP 3 dissolved in dichloromethane with different concentrations [8]

Set different concentrations of CP **2** and CP **3** which dissolved by CH_2Cl_2, then detected these samples by ultraviolet spectrophotometer. The results are shown in Fig S8-1 and Fig S8-2.

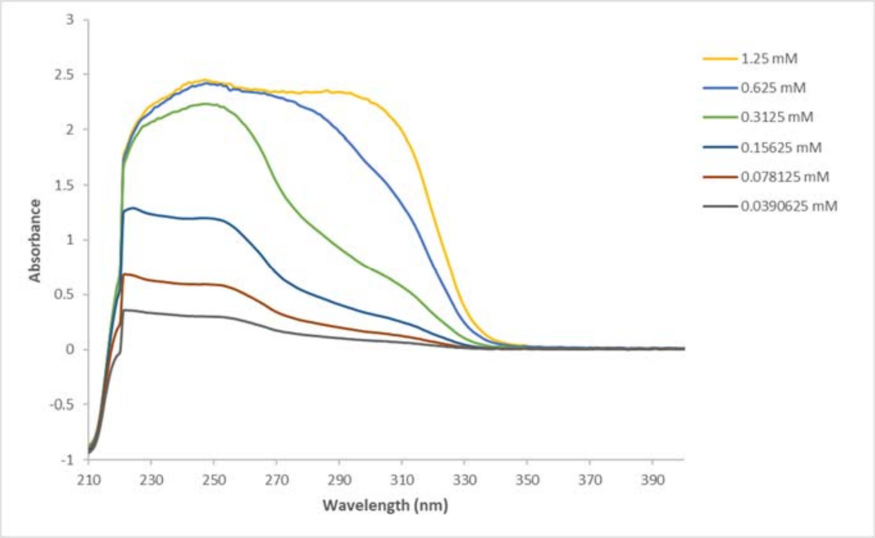

Figure S8-1 The UV-vis spectra of CP **2** dissolved in CH_2Cl_2 with different concentrations

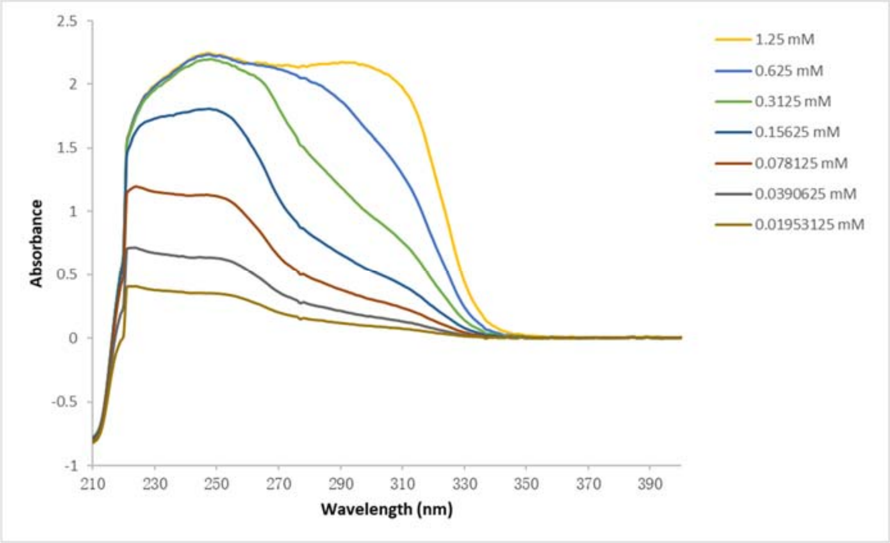

Figure S8-2 The UV-vis spectra of CP **3** dissolved in CH_2Cl_2 with different concentrations

9. Procedures for OH^- transport experiments[9]

9.1 Preparation of large unilamellar vesicles (LUVs)

Egg yolk L-α-phosphatidylcholine (EYPC, 15 mg) was dissolved in $CHCl_3$ (5.0 mL). The solution was evaporated under reduced pressure and the resulting thin film was further dried under high vacuum at 55 ºC for 3 h. Then the lipid film was hydrated with HEPES buffer solution which contains HPTS fluorescein (1.5 mL, HEPES (10 mM), HPTS (0.1 mM), NaCl (100 mM), pH= 7.0) at 40 ºC for 2 h to give a milky suspension. The resulting suspension was subjected to ten freeze-thaw cycles by using liquid N_2 to freeze and warm water bath (60 ºC) to thaw. The suspension was extruded through hydrophilic cellulose ester membranes (FilterBio, 0.22 μm) for six times and dialyzed with membrane tube (MWCO = 14000) against the same HEPES buffer solution (500 mL, without HPTS) for three times at 5 ºC to remove un-entrapped HPTS and produce LUVs suspension ([lipid] = 13.3mM).

9.2 Fluorescent experiments

Fluorescent experiments were measured on a fluorospectrophotometer F380 (TIANJIN GANGDONG SCI.&TECH.DEVELOPMENT CO,.LTD). The typical procedure was described as follows. HEPES buffer solution (2.0 mL, HEPES (10 mM), KCl (100 mM), pH= 7.6) was placed in a fluorimetric cuvette. To the cuvette, the prepared vesicle suspension (13.3 mM, 100 μL) and CP **2** or CP **3** in DMF (5.0 mM, 20.0 μL) were added with gentle stirring. The fuorescent intensity (I_t) was continuously monitored at 510 nm (excitation at 460 nm) in 20 min. Then, Triton X-100 aqueous solution (50%, 40 μL) was added with gentle stirring. The intensity was monitored until the fluorescent intensity (I_∞) did not change. The collected data were then normalized into the fractional change in fluorescence given by $(I_t-I_0)/(I_\infty-I_0)$, where I_0 is the initial intensity.

10. Procedures for H^+ transport experiments[9]

10.1 Preparation of large unilamellar vesicles (LUVs)

Egg yolk L-α-phosphatidylcholine (EYPC, 15 mg) was dissolved in $CHCl_3$ (5.0 mL). The solution was evaporated under reduced pressure and the resulting thin film was further dried under high vacuum at 55 ºC for 3 h. Then the lipid film was hydrated with HEPES buffer solution which contains HPTS fluorescein (1.5 mL, HEPES (10 mM), HPTS (0.1 mM), KCl (100 mM), pH= 7.0) at 40 ºC for 2 h to give a milky suspension. The resulting suspension was subjected to ten freeze-thaw cycles by using liquid N_2 to freeze and warm water bath (60 ºC) to thaw. The suspension was extruded through hydrophilic cellulose ester membranes (FilterBio, 0.22 μm) for six times and dialyzed with membrane tube (MWCO = 14000) against the same HEPES buffer solution (500 mL, without HPTS) for three times at 5 ºC to remove un-entrapped HPTS and produce LUVs suspension ([lipid] = 13.3mM).

10.2 Fluorescent experiments

Fluorescent experiments was measured on a fluorospectrophotometer F380 (TIANJIN GANGDONG SCI.&TECH.DEVELOPMENT CO,.LTD). The typical procedure was described as follows. HEPES buffer solution (2.0 mL, HEPES (10 mM), NaCl (100 mM), pH= 5.5) was placed in a fluorimetric cuvette. To the cuvette, the prepared vesicle suspension (13.3 mM, 100 μL) and CP **2** or CP **3** in DMF (5.0 mM, 20.0 μL) were added with gentle stirring. The fuorescent intensity (I_t) was continuously monitored at 510 nm (excitation at 460 nm) in 20 min. Then, Triton X-100 aqueous solution (50%, 40 μL) was added with gentle stirring. The intensity was monitored until the fluorescent intensity (I_∞) did not change. The collected data were then normalized into the fractional change in fluorescence given by $(I_t\text{-}I_0)/(I_\infty\text{-}I_0)$, where I_0 is the initial intensity.

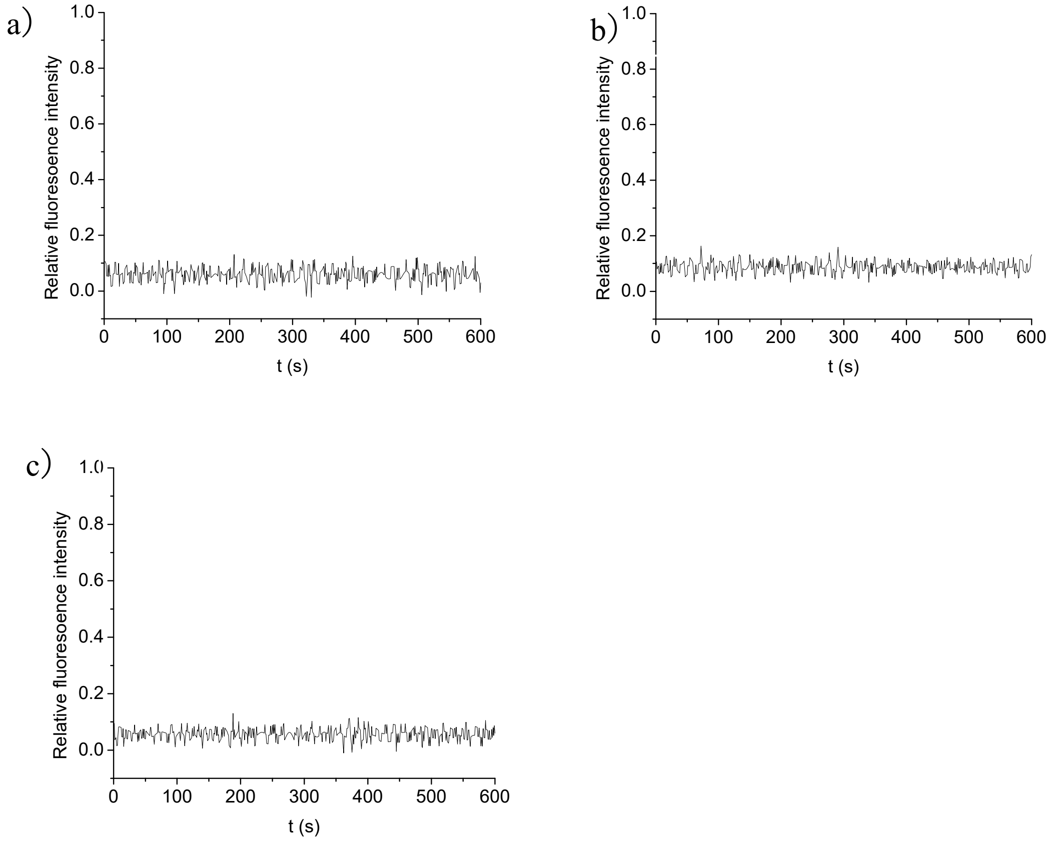

Figure S10-1 Change in fluorescent intensity of vesicle against time after addition of pure (a) DMF, (b) **2** and (c) **3** (all buffered to pH 5.5).

11. Gels images of CP 2 and 3 assemblies

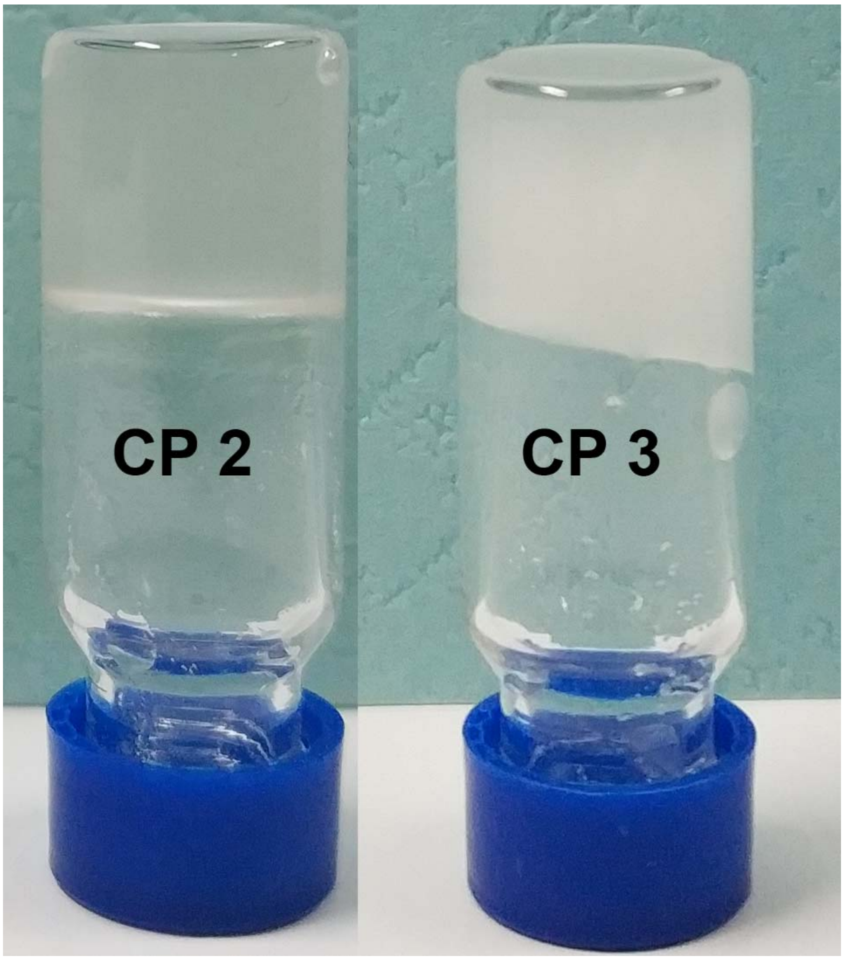

Figure S11. The images of the gels formed by CP 2 and 3.

12. SEM and TEM images

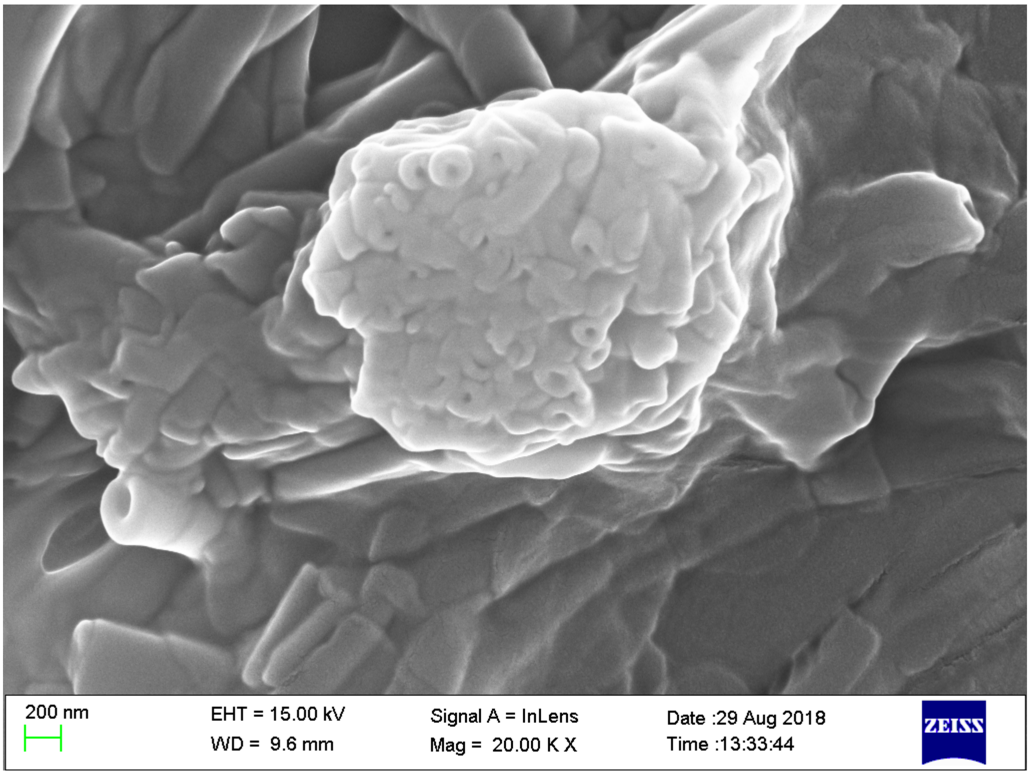

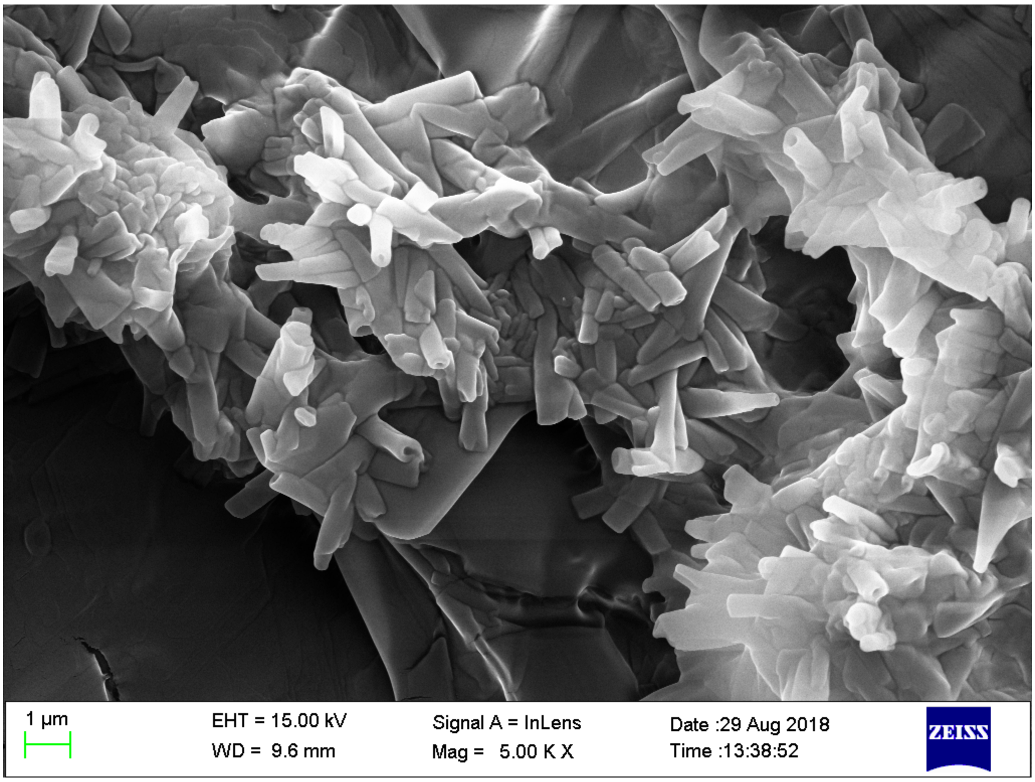

Figure S12-1 The SEM images of gels formed by **2.**

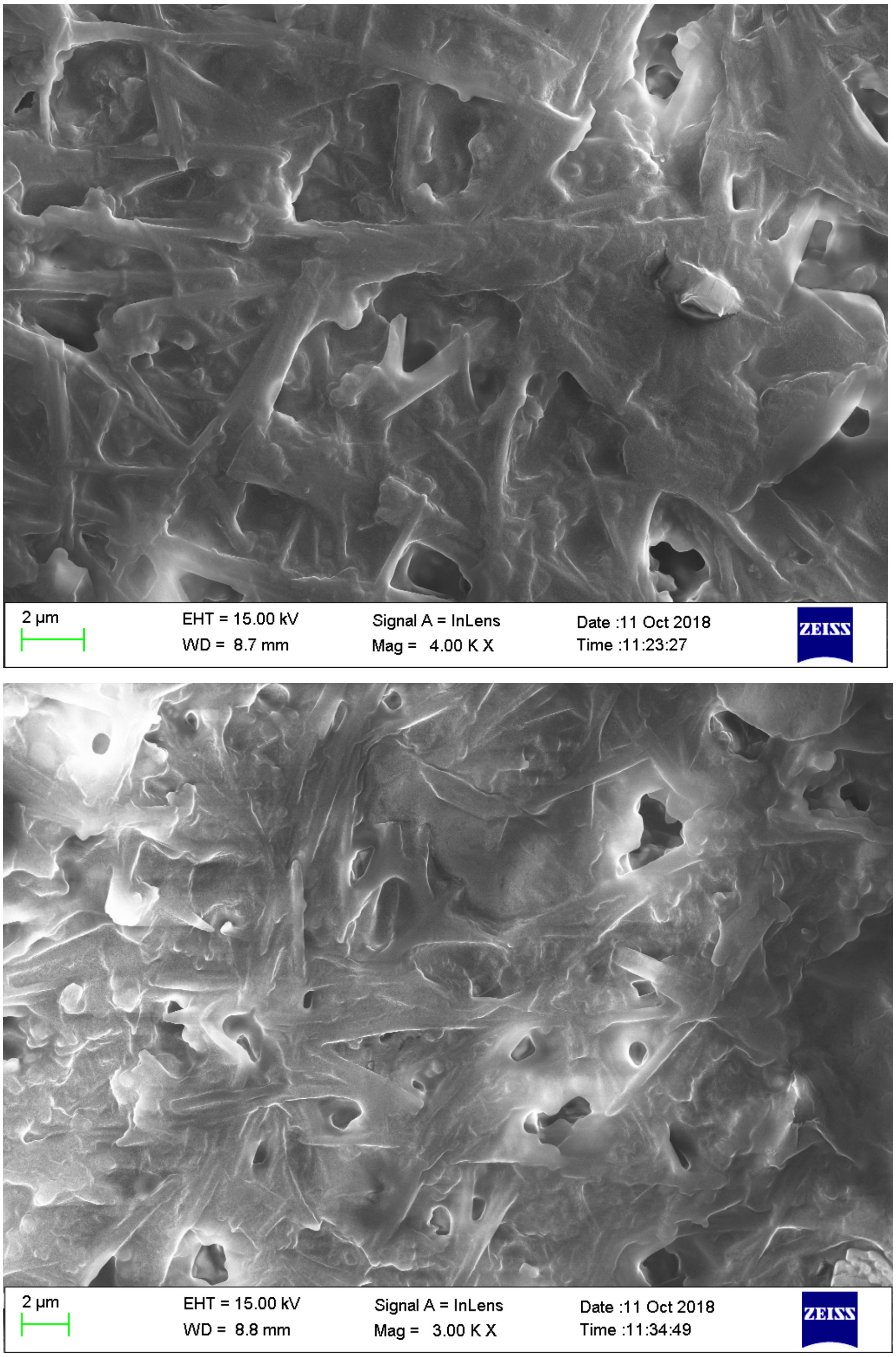

Figure S12-2 The SEM images of gels formed by **3.**

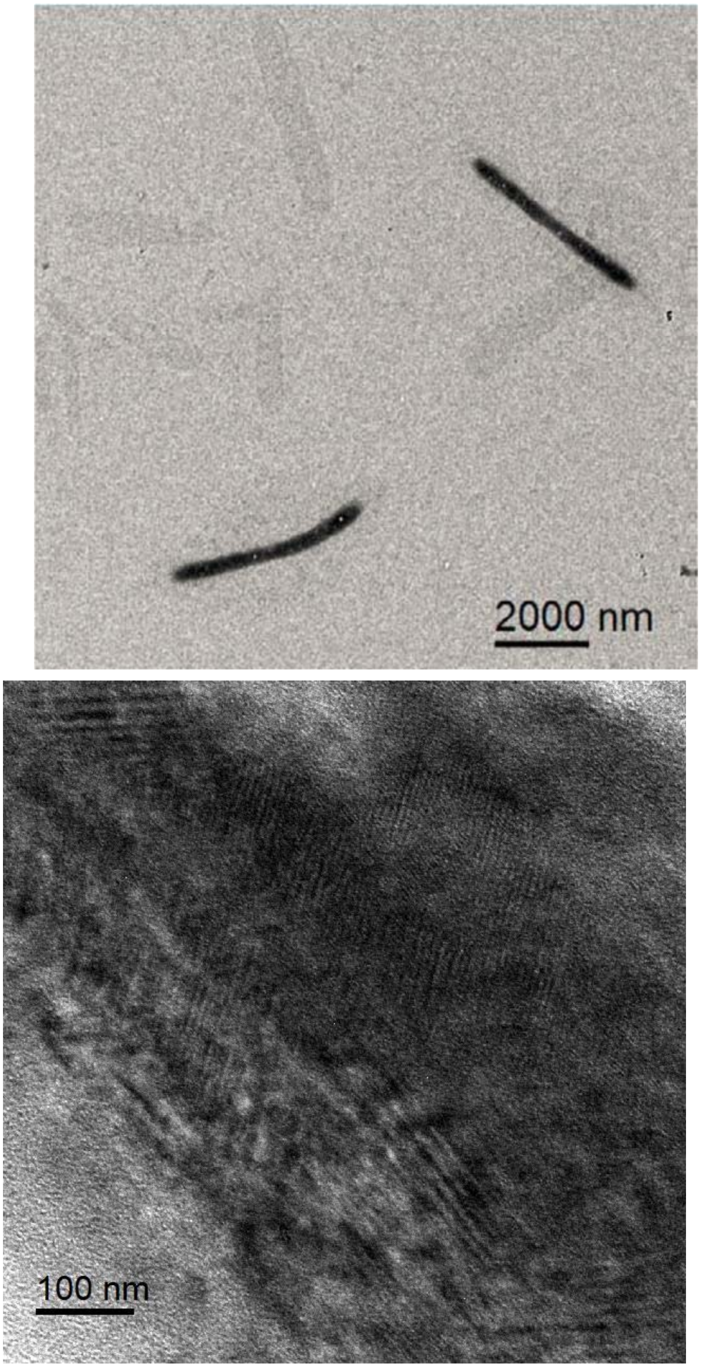

Figure S12-3 The TEM images of gels formed by **2** (dispersed in n-hexane)**.**

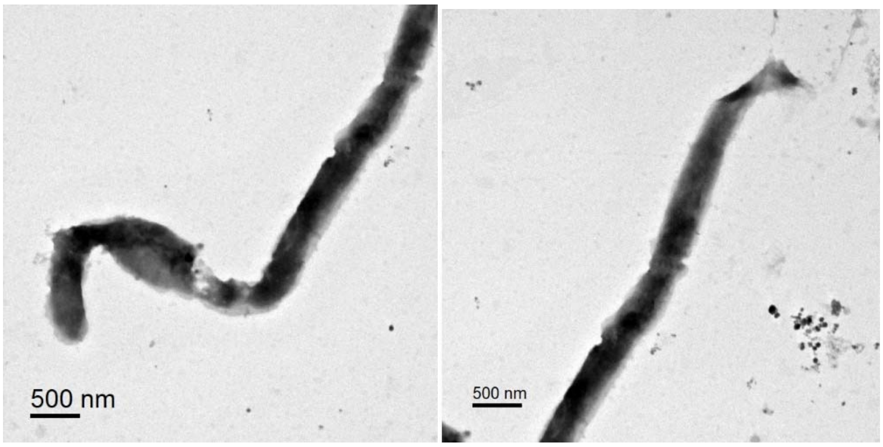

Figure S12-4. The TEM images of gels formed by CP **3** (dispersed in n-hexane) **.**

13. DFT calculations

To probe the conformations of the CPs, we do DFT calculations on the CP **2** and CP **3**. The calculations were carried out with the GAUSSIAN09 package. Due to the side chain of CPs having no influence on the conformation of the structures, we cut off the side chain of CPs to simplify the calculation. Firstly, the conformational searches of CP **2** showed that two structures were stable within 3 kcal/mol at M052x/6-31g(d) level. One of the conformers showed planar like structure which was familiar to form nanotubes by stacking on each other face to face. Thus, the reasonable dimers of CP **2** via parallel or antiparallel β -sheet-like hydrogen bonding were calculated like our researches before.

In the gas phase the BSSE-corrected binding energies calculated with the M05-2X/6-31G + (d,p) method are −31.4 and −20.30 kcal/mol for the parallel and antiparallel dimers of CP **2**, respectively. To dimers of CP **3**, the parallel and antiparallel binding energies are -51.2 and -40.2 kcal/mol, respectively. All the results suggest that parallel dimer is more stable than the antiparallel one, because the latter forms weaker hydrogen bonds judging from hydrogen-bonding distances and angles and also suffers from a geometrical deformation. Inclusion of the solvent effects give the same results. The results suggest that the parallel stacking modes of the cyclic γ-peptide are more energetically favourable than the antiparallel, and the antiparallel dimer of **2** is not reachable due to low stability in solvent. However, the CP **3** can both form the dimer with the antiparallel and parallel stacking modes, respectively. (Tabe S13-1)

Table S13-1. The calculated interaction, dimerization and deformation energies for dimers. [a]

	ΔE	ΔE_{sol}
**antiparallel dimer of 2**	-32.60 (-20.30)	0.56
**parallel dimer of 2**	-45.44 (-31.40)	-10.44
**antiparallel dimer of 3**	-50.20 (-40.20)	-18.36
**parallel dimer of 3**	-62.52 (-51.20)	-29.83

[a] Energies in kcal/mol; values in parentheses showed the corrected BSSE energies; ΔE for the binding energies; ΔE_{inter} for the interaction energies; ΔE_{sol} for the binding energies in $CHCl_3$.

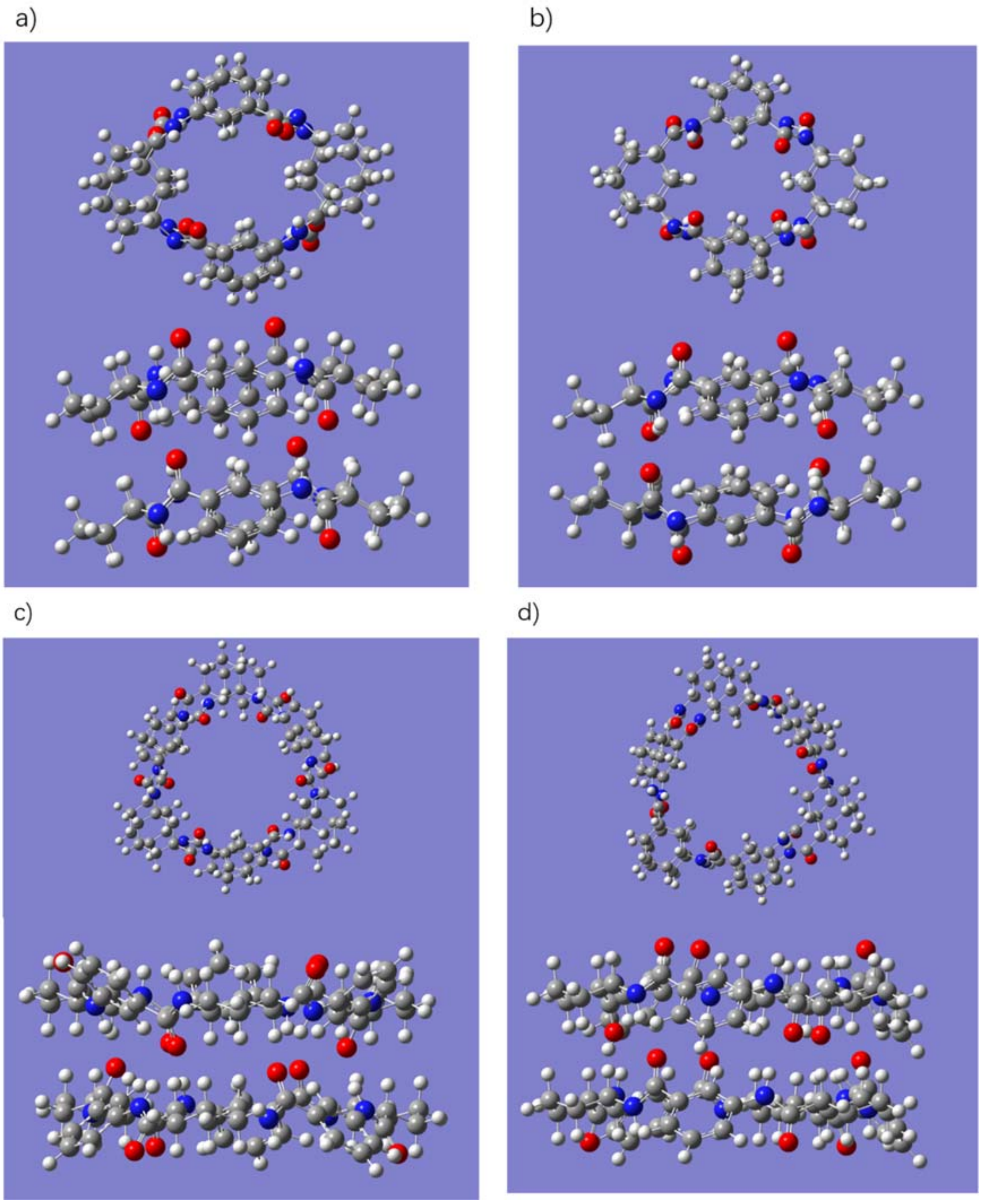

Figure S13-1. The top view (top) and side view (bottom) of optimized geometries of a) parallel dimer of **2**; b) antiparallel dimer of **2**; c) parallel dimer of **3**; d) antiparallel dimer of **3**. (the side chains are all cut off for simplified model)

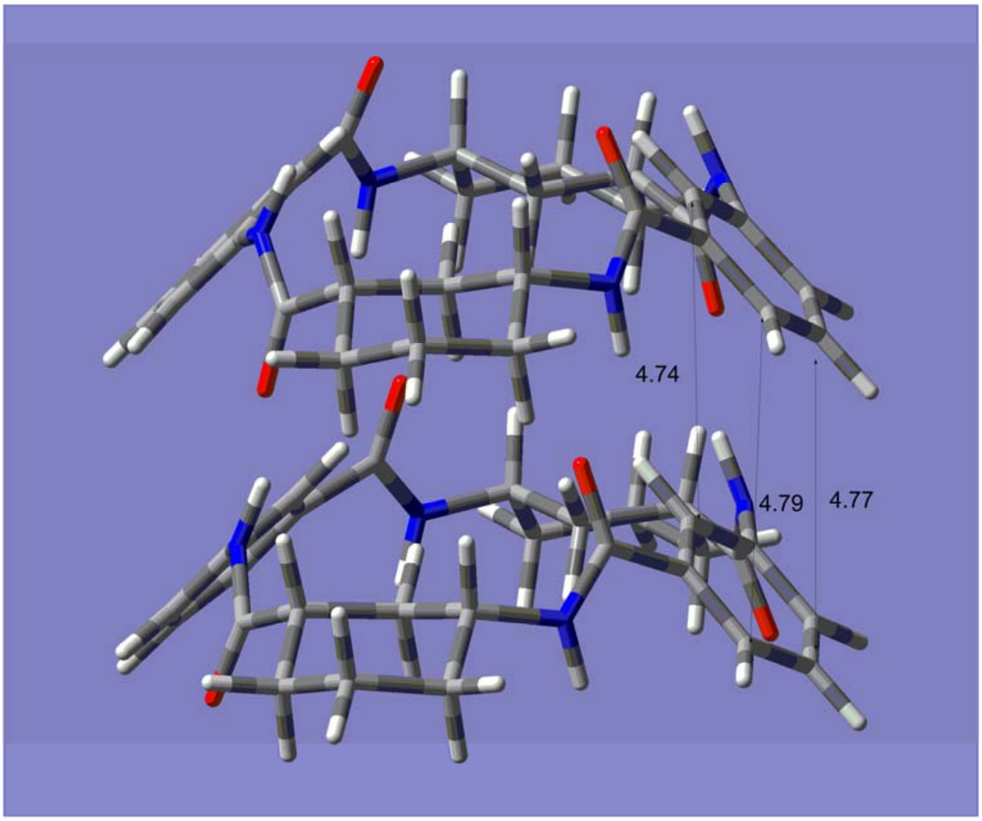

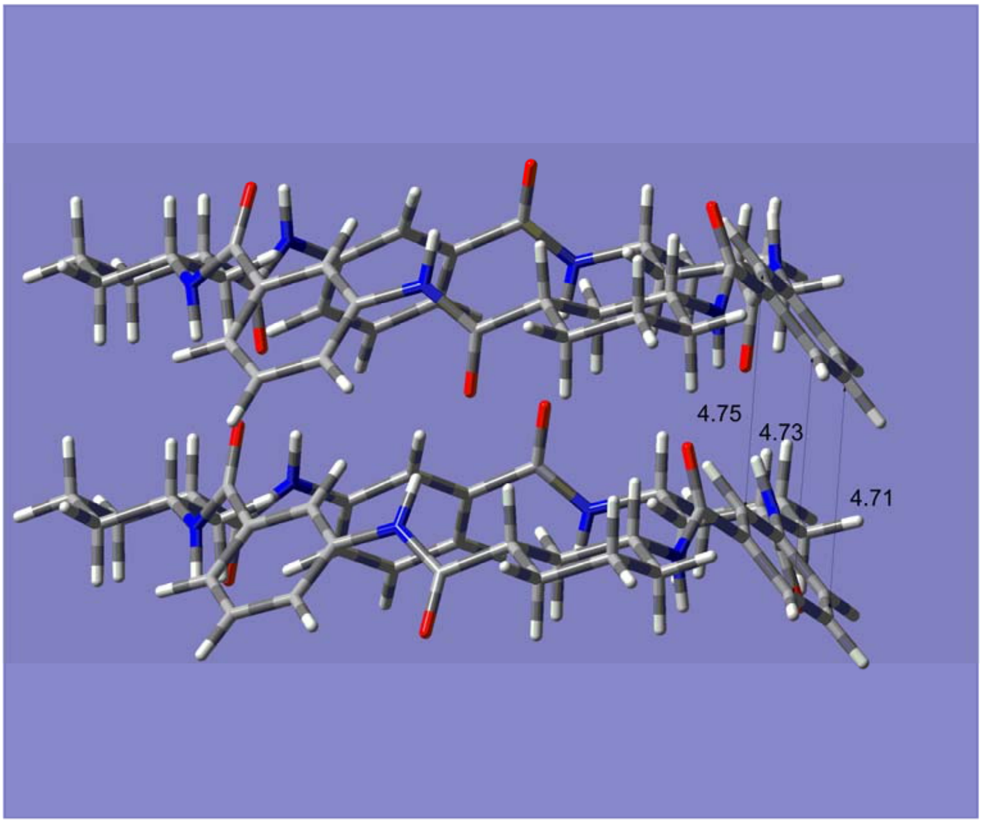

Figure S13-2. The distances between the benzene rings in the parallel stacking conformation of dimers formed by **2** (top) and **3** (bottom). Values for selected interatomic distance are given in Å.

References：

[1] Y. Hu, S. L. Yu, Y. J. Yang, J. Zhu and J. G. Deng, Chin. J. Chem., 2006, 24, 795-799.

[2] Yang, Qinglai, et al. "Dynamic covalent diblock copolymers: instructed coupling, micellation and redox responsiveness." Macromolecules 47.21 (2014): 7431-7441.

[3] Brea, R.; Castedo, L.; Granja, J. R., Large-diameter self-assembled dimers of α,γ-cyclic peptides, with the nanotubular solid-state structure of cyclo-[(L-Leu-D-^{Me}N-γ-Acp)$_4$-]•4$CHCl_2COOH$. Chem. Commun. 2007, 3267-3269.

[4] Amorín, M.; Castedo, L.; Granja, J. R., Folding Control in Cyclic Peptides through N-Methylation Pattern Selection: ormation of Antiparallel β-Sheet Dimers, Double Reverse Turns and Supramolecular Helices by 3α, γ Cyclic Peptides. Chem. Eur. J. 2008, 14, 2100-2111.

[5] Haris, P. I.; Chapman, D., The conformational analysis of peptides using fourier transform IR spectroscopy. Biopolymers 1995, 37, 251-263.

[6] Jagannadh, B.; Reddy, M. S.; Rao, C. L.; Prabhakar, A.; Jagadeesh, B.; Chandrasekhar, S., Self-assembly of cyclic homo- and hetero-β-peptides with cis-furanoid sugar amino acid and β-hGly as building blocks. Chem. Commun. 2006, 4847-4849.

[7] Li, L.; Zhan, H.; Duan, P.; Liao, J.; Quan, J.; Hu, Y.; Chen, Z.; Zhu, J.; Liu, M.; Wu, Y.-D.; Deng, J., Self-Assembling Nanotubes Consisting of Rigid Cyclic γ-Peptides. Adv. Funct. Mater. 2012, 22, 3051-3056.

[8] Guha, S.; Drew, M. G. B.; Banerjee, A., Dipeptide Nanotubes, with N-Terminally Located ω-Amino Acid Residues, That are Stable Proteolytically, Thermally, and Over a Wide Range of pH. Chem. Mater. 2008, 20, 2282-2290.

[9] Traikia, M.; Warschawski, D. E.; Recouvreur, M.; Cartaud, J.; Devaux, P. F. Eur. Biophys. J. 2000, 29, 184-195.
